# Unifying Long-Term Plasticity Rules for Excitatory Synapses by Modeling Dendrites of Cortical Pyramidal Neurons

**DOI:** 10.1016/j.celrep.2019.11.068

**Published:** 2019-12-24

**Authors:** Christian Ebner, Claudia Clopath, Peter Jedlicka, Hermann Cuntz

**Affiliations:** 1 Frankfurt Institute for Advanced Studies, 60438 Frankfurt am Main, Germany; 2 Ernst Strüngmann Institute (ESI) for Neuroscience in Cooperation with Max Planck Society, 60528 Frankfurt am Main, Germany; 3 NeuroCure Cluster of Excellence, Charité–Universitätsmedizin Berlin, 10117 Berlin, Germany; 4 Institute for Biology, Humboldt-Universität zu Berlin, 10117 Berlin, Germany; 5 Computational Neuroscience Laboratory, Bioengineering Department, Imperial College London, London SW7 2AZ, UK; 6 Institute of Clinical Neuroanatomy, Neuroscience Center, Goethe University Frankfurt, 60528 Frankfurt am Main, Germany; 7 ICAR3R–Interdisciplinary Centre for 3Rs in Animal Research, Faculty of Medicine, Justus-Liebig-University, 35392 Giessen, Germany

**Keywords:** compartmental modeling, synaptic plasticity, spike timing dependent plasticity (STDP), dendritic spikes, NMDA spikes, long term potentiation (LTP), long term depression (LTD), synaptic cooperativity

## Abstract

A large number of experiments have indicated that precise spike times, firing rates, and synapse locations crucially determine the dynamics of long-term plasticity induction in excitatory synapses. However, it remains unknown how plasticity mechanisms of synapses distributed along dendritic trees cooperate to produce the wide spectrum of outcomes for various plasticity protocols. Here, we propose a four-pathway plasticity framework that is well grounded in experimental evidence and apply it to a biophysically realistic cortical pyramidal neuron model. We show in computer simulations that several seemingly contradictory experimental landmark studies are consistent with one unifying set of mechanisms when considering the effects of signal propagation in dendritic trees with respect to synapse location. Our model identifies specific spatiotemporal contributions of dendritic and axo-somatic spikes as well as of subthreshold activation of synaptic clusters, providing a unified parsimonious explanation not only for rate and timing dependence but also for location dependence of synaptic changes.

## Highlights

• A phenomenological synaptic plasticity rule is applied to a pyramidal neuron model
• Model reproduces rate-, timing-, and location-dependent plasticity results
• Active dendrites allow plasticity via dendritic spikes and subthreshold events
• Cooperative plasticity exists across the dendritic tree and within single branches

## Graphical Abstract

**Figure:**
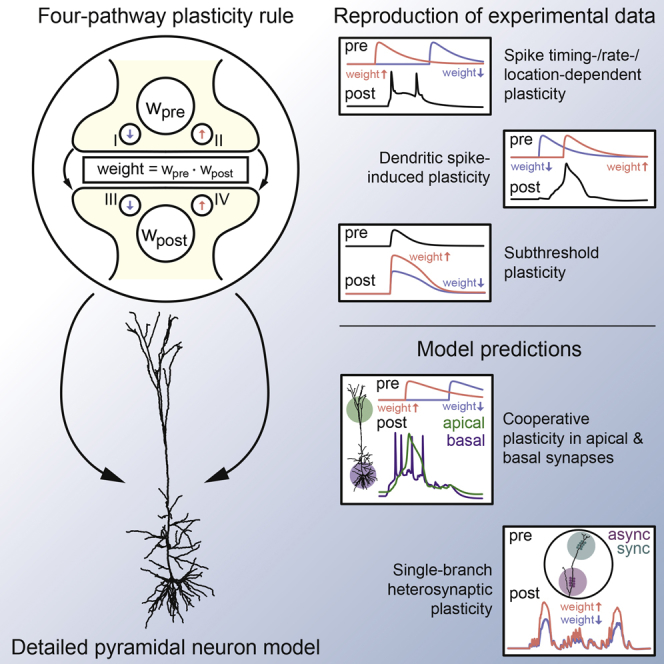

## Teaser

Synaptic plasticity is shaped by local dynamic processes within dendritic trees. Ebner et al. present a biologically inspired plasticity rule and study its implications in a detailed model of a pyramidal cell. They provide a unified description of rate, timing, and location dependence and predict cooperative plasticity in dendrites.

## Introduction

Adaptive behavior, guided by learning and memory processes, can be seen as a macroscopic manifestation of microscopic long-term changes in synaptic strength (Bliss and Collingridge, 1993). Such changes have been proposed to be related to the causal contribution of a presynaptic (pre) cell to the excitation of a postsynaptic (post) cell according to Hebbian theory (Hebb, 1949). Thus, a number of studies exploring various “spike timing-dependent plasticity” (STDP) (Abbott and Nelson, 2000) protocols have investigated the relationship of the precise timing between presynaptic and postsynaptic action potentials (APs) on the efficacy of synapses. In the simplest arrangement, pre-APs preceding post-APs (pre-post, positive timing) by a few milliseconds typically result in synaptic long-term potentiation (LTP), whereas the opposite order (post-pre, negative timing) leads to long-term depression (LTD) (Bi and Poo, 1998, Markram et al., 1997). However, each newly tested plasticity protocol has led to the discussion of new parameters. In particular, when bursts of APs are considered, the frequency of these bursts heavily influences the results of the simple STDP concept. Higher frequencies tend to increase the strength of LTP at positive timings (Markram et al., 1997, Sjöström et al., 2001) and sometimes even convert LTD at negative timings into LTP, bypassing the pre-post timing requirement (Sjöström et al., 2001). Also, the location of synapses along the dendritic tree was shown to play an important role, with LTD often becoming more prominent in distal synapses (Froemke et al., 2005, Sjöström and Häusser, 2006), a likely consequence of voltage attenuation of backpropagating action potentials (bAPs) in dendrites (Stuart et al., 1997), where LTP was recovered by boosting bAPs through dendritic current injection or cooperative synaptic inputs (Sjöström and Häusser, 2006). In addition to these effects of frequency and location on STDP, plasticity can also be induced by depolarization that originates from other sources besides bAPs in the postsynaptic neuron, e.g., dendritic Ca^2+^ spikes (Golding et al., 2002, Kampa et al., 2006, Letzkus et al., 2006), *N*-methyl-D-aspartate (NMDA) spikes (Brandalise et al., 2016, Gordon et al., 2006), or excitatory postsynaptic potentials (EPSPs) alone (Sandler et al., 2016, Weber et al., 2016). For all these reasons, the concept of classical STDP as a self-contained mechanism has been debated (Clopath and Gerstner, 2010, Clopath et al., 2010, Goldberg et al., 2002, Lisman and Spruston, 2005, Shouval et al., 2010). It stands to reason that the principle of STDP is only one manifestation of an underlying general plasticity framework (Feldman, 2012, Shouval et al., 2010). In that case, the question emerges as to which biophysical pathways contribute to the results from classical STDP protocols and in which ways they are related to other plasticity protocols. A large number of theories and models have been developed with both phenomenological (Morrison et al., 2008) as well as biophysical (Graupner and Brunel, 2010) backgrounds that explore these questions, but only a few have recently proposed a unifying concept of multiple pre- and postsynaptic plasticity pathways (Costa et al., 2015) in neuron models with extended dendrites (Bono and Clopath, 2017, Kastellakis et al., 2016, Krieg and Triesch, 2014, Solinas et al., 2019, Urbanczik and Senn, 2014).

Although many biophysical details of excitatory long-term synaptic plasticity are still not fully understood, it is widely accepted that postsynaptic Ca^2+^ plays a fundamental role. According to some theories and experiments, low levels of Ca^2+^ lead to no changes in synaptic strength, whereas intermediate levels cause LTD and high levels lead to LTP (Artola and Singer, 1993, Artola et al., 1990, Graupner and Brunel, 2012, Lisman, 1989, Shouval et al., 2002). However, more recent experiments have indicated that the levels of postsynaptic Ca^2+^ by themselves are not always good predictors for plasticity (Nevian and Sakmann, 2006), and increasing evidence suggests that multiple partly independent signaling routes that use Ca^2+^ exist (Bender et al., 2006, Jedlicka and Deller, 2017, Oliet et al., 1997, Sjöström et al., 2003, Sjöström et al., 2007), ultimately leading to a mixture of synaptic changes both expressed at presynaptic and postsynaptic sites (Sjöström et al., 2007). In our phenomenological plasticity model, we incorporated four signaling routes that are loosely related to signaling routes in long-term synaptic plasticity that have been characterized previously. Our plasticity model is based on and extends an existing phenomenological voltage-dependent STDP rule (Clopath and Gerstner, 2010, Clopath et al., 2010). We show in our simulations that a single, dendritic-location-independent plasticity mechanism is able to reconcile many of the differences found in experiments, including plasticity measurements that previous models were not able to account for. We propose, in line with previous suggestions (Feldman, 2012, Shouval et al., 2010), that concepts such as the Ca^2+^ level hypothesis mentioned above and classical STDP rules could all be consequences of the same pathways that strongly depend on local interactions at the synapse.

## Results

### A Plasticity Rule Based on Pre- and Postsynaptic Pathways

In our plasticity model, we introduced four pathways that contributed to changes in both pre- and postsynaptic weight factors. Although implemented as a phenomenological rule, its mechanisms were inspired by well-established biophysical pathways described in a multitude of experimental studies on long-term synaptic plasticity.

**Figure 1:**
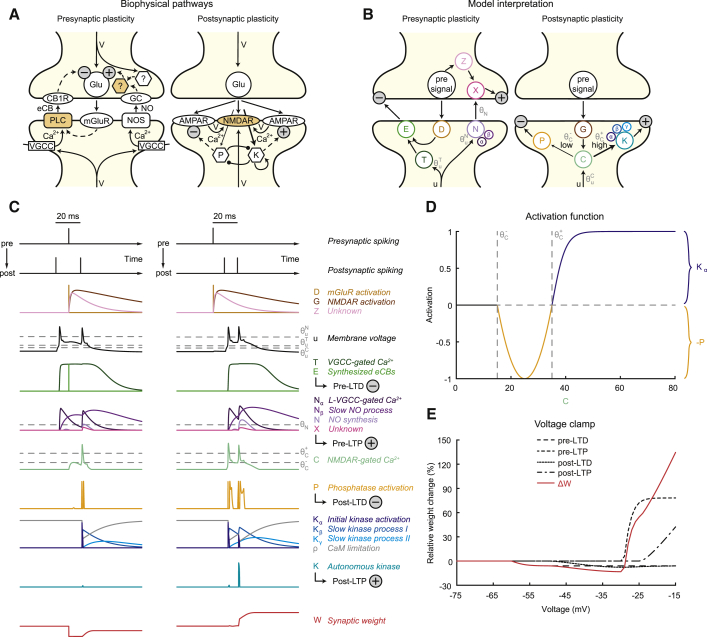
A Plasticity Rule Based on Separate Pathways for Pre- and Postsynaptic Plasticity (A) Simplified illustration of the biophysical pathways that inspired our model. Pre-LTD (left) is induced if postsynaptic Ca^2+^ influx through VGCCs coincides with an mGluR-mediated signaling cascade, causing eCB release by PLC and subsequent downregulation of transmitter release probability (minus sign) by CB1Rs. Pre-LTP (left) reportedly requires Ca^2+^ influx through VGCCs, triggering NO synthesis, which is then detected by GC and integrated with a presynaptic signal by an unknown presynaptic coincidence detector to increase release probability (plus sign). Post-LTD and post-LTP (right) are driven by coincident depolarization and activation of NMDARs. Lower amounts of NMDAR-gated Ca^2+^ (thin arrow) activate phosphatases (P), causing a reduction of AMPAR efficacy (minus sign). Higher amounts of NMDAR-gated Ca^2+^ (bold arrow) activate kinases (K), causing an increase in AMPAR efficacy (plus sign). Coincidence detectors of pre- and postsynaptic activity are indicated with orange color. (B) Model interpretation and abstraction of the biophysical pathways in (A). Pre-LTD (left) is dependent on the coincidence between the presynaptic signal D and a postsynaptic signal based on membrane voltage, reflected in the variable T. The resulting trace E indicates the amount of pre-LTD (minus sign). Pre-LTP (left) requires coincidence of a presynaptic signal Z and a postsynaptic trace N (based on membrane voltage by $N_{\alpha }$ and $N_{\beta }$). The amount of pre-LTP (plus sign) is indicated by X. Post-LTD and post-LTP (right) depend on coincidence between the presynaptic signal G and a portion of membrane voltage u. If the resulting trace C reaches lower levels, P is activated, indicating post-LTD (minus sign), whereas higher levels activate K, indicating post-LTP (plus sign). (C) Traces computed by the plasticity rule for two sample stimulation patterns (see top): post-pre-post pairing (left column) and pre-post-post pairing (right column). The various rows include all fundamental variables of the plasticity rule with color code from (B). Overall synaptic weight W is shown in the bottom row in red. Loose analogies of the model’s variables to biophysical processes are given in italic type to the right. (D) Transfer function for post-LTD and post-LTP. Activation of either −P or $K_{\alpha }$ is shown as a function of C. (E) Voltage clamp simulation while a single presynaptic event is evoked. As a function of clamped voltage, absolute contribution of each of the four pathways is plotted (black lines), as well as the overall relative weight change (red line). See also Figure S1.

Briefly, presynaptically expressed LTD (pre-LTD; Figure 1A, left) occurs when metabotropic glutamate receptors (mGluRs) and postsynaptic voltage-gated Ca^2+^ channels (VGCCs) are activated simultaneously (Heifets and Castillo, 2009). Phospholipase C (PLC) then integrates these two signals in the process of synthesizing endocannabinoids (eCBs) (Hashimotodani et al., 2005), which retrogradely act on presynaptic type 1 cannabinoid receptors (CB1Rs) to reduce transmitter release probability (Heifets and Castillo, 2009), causing pre-LTD. Presynaptically expressed LTP (pre-LTP) is thought to occur when postsynaptic L-type VGCCs (L-VGCCs) are activated, presumably triggering synthesis of nitric oxide (NO) (Padamsey et al., 2017, Pigott and Garthwaite, 2016), possibly by calmodulin (CaM) at nitric oxide synthases (NOSs) (Abu-Soud et al., 1994). NO retrogradely acts on presynaptic guanylyl cyclase (GC) (Koesling et al., 2004), triggering a signaling chain that is combined with a presynaptic signal by a presynaptic coincidence detector that has yet to be discovered (Padamsey et al., 2017). Postsynaptically expressed LTD and LTP (post-LTD/-LTP; Figure 1A, right) are described as both being driven by coincident binding of glutamate and depolarization of postsynaptic NMDA receptors (NMDARs) (Lüscher and Malenka, 2012). Strong NMDAR-gated Ca^2+^ influx activates protein kinases, such as Ca^2+^/CaM-dependent protein kinase II (CaMKII), whereas weak Ca^2+^ influx activates their counterpart molecules, protein phosphatases such as protein phosphatase 1 and calcineurin (Lisman, 1989). Kinases increase and phosphatases decrease synaptic efficacy determined by α-amino-3-hydroxy-5-methyl-4-isoxazolepropionic acid receptors (AMPARs), essentially forming complementary mechanisms of postsynaptic LTD and LTP induction (Lüscher and Malenka, 2012). Studies indicate that CaMKII is able to phosphorylate itself (autophosphorylation) due to its specific subunit structure and that this process is more likely to take effect if pulses of Ca^2+^ bound to CaM are applied rapidly (De Koninck and Schulman, 1998), suggesting that this mechanism could play a role in the frequency dependence of plasticity.

In our model, pre-LTD (indicated by the variable E; Figure 1B, left; see also Figure S1) was induced when the low-pass-filtered postsynaptic voltage trace T coincided with the brief presynaptic signal D. Due to the transient nature of D, pre-LTD was only induced if the postsynaptic cell experienced depolarization shortly before the presynaptic signal, e.g., if the stimulation included a post-pre pair (Figure 1C, medium green color in left versus right column). Consequently, this mechanism only detected post-pre timings and was insensitive to pre-post timings, consistent with pre-LTD in experimental studies (Nevian and Sakmann, 2006, Sjöström et al., 2003). Pre-LTP (indicated by the variable X; Figure 1B, left) in our model required that two consecutively filtered traces based on postsynaptic voltage $N_{\alpha }$ and $N_{\beta }$ coincided to result in a trace N. Owing to $N_{\beta }$ being filtered from $N_{\alpha }$, N was sensitive to the frequency of postsynaptic events during postsynaptic activity due to summation (Figure 1C, light violet color). Only if N was sufficiently elevated during the occurrence of the slow presynaptic signal Z, pre-LTP was switched on. Post-LTD (indicated by the variable P; Figure 1B, right) was modeled by calculating the coincidence of the slow presynaptic signal G and a portion of membrane voltage u. The resulting variable C was subjected to an activation function with two thresholds, namely, $\theta_{C}^{-}$ and $\theta_{C}^{+}$ (Figure 1D). This formalism was consistent with Ca^2+^-level-based rules (Artola and Singer, 1993, Lisman, 1989, Shouval et al., 2002) that have previously been used for modeling synaptic plasticity (see Discussion). No synaptic weight changes were induced below $\theta_{C}^{-}$. Whenever C resided between $\theta_{C}^{-}$ and $\theta_{C}^{+}$, post-LTD was activated (Figure 1C, orange color). To model post-LTP (indicated by the variable K; Figure 1B, right), the portion of C above $\theta_{C}^{+}$, named $K_{\alpha }$, was used to compute the two slower traces $K_{\beta }$ and $K_{\gamma }$, which were filtered versions of $K_{\alpha }$. The variable ρ limited the sum of $K_{\alpha }$ and $K_{\beta }$. Post-LTP was only switched on when $K_{\alpha }$, $K_{\beta }$, and $K_{\gamma }$ were nonzero. Thus, similarly to pre-LTP, this mechanism was frequency dependent. A pre-post-post protocol, therefore, evoked considerably more post-LTP than a post–pre-post protocol (Figure 1C, turquoise color in right versus left column).

The rule’s voltage dependence is demonstrated in Figure 1E. One single presynaptic event was evoked while the postsynaptic cell was clamped to values between −75 mV and −15 mV. Voltages below −60 mV led to no change in weight, whereas voltages between −60 mV and about −28 mV caused LTD and voltages above that caused net LTP. Consistent with experiments on voltage dependence of LTD pathways (Oliet et al., 1997), post-LTD more strongly depended on depolarization than pre-LTD. To consider all the effects of realistic firing behavior, active dendrites, and synapse location, we incorporated our plasticity model into a highly detailed cortical layer 5b (L5b) pyramidal cell model (Hay et al., 2011).

### Effects of Synapse Location on Rate- and Timing-Dependent Plasticity

**Figure 2:**
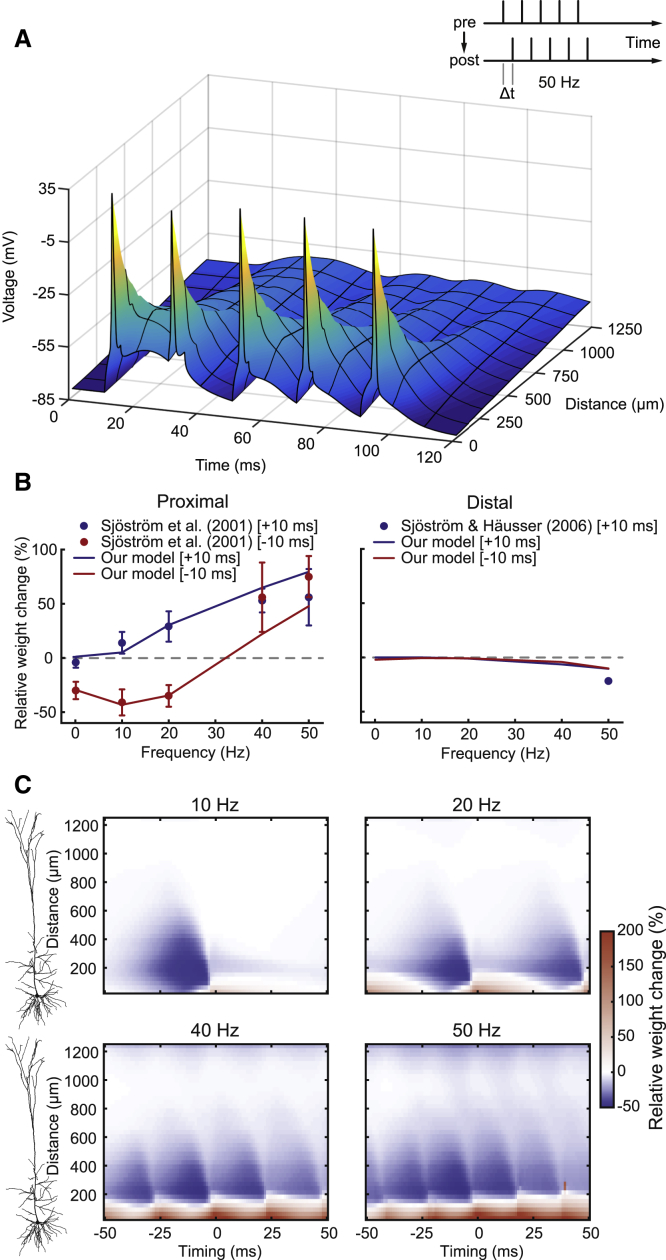
Effects of Synapse Location on Rate- and Timing-Dependent Plasticity (A) Stimulation protocol (Sjöström et al., 2001) (inset) and postsynaptic voltage profile of the neuron model with five somatic spikes (50 Hz) starting at time 10 ms. Voltage profile is shown as a function of time and location along one specific path from the soma into the apical dendrite. (B) Model results as relative weight changes (lines) when fit to experimental data (filled circles, mean ± SEM) at a proximal location (90 μm in the model; left) and at a distal location (669 μm in the model; right) for different intra-burst frequencies. Pre- and postsynaptic bursts were shifted by either +10 ms (blue) or −10 ms (red). Experimental data was recreated from Sjöström et al. (2001) Figure 1D and Figure 7B and from Sjöström and Häusser (2006) Figure 3 (by using the exponential fit of inset data). (C) Spatiotemporal plasticity windows showing relative weight changes (color coded) as a function of burst timing (x axis) and distance from the soma (y axis; see cell morphology on the left). Synaptic weight changes were calculated at 41 different locations and 101 different timings. See also Figure S2.

In the first stimulation protocol we used, regular bursts of five pre- and five postsynaptic APs were evoked at either pre-post (Δt=+10ms) or post-pre (Δt=−10ms) timings (Figure 2A inset; see STAR Methods). The frequency within the bursts (intra-burst frequency) varied between 0.1 Hz and 50 Hz. Even at 50 Hz, distal dendrites in the neuron model experienced only weak depolarization due to bAP attenuation (Figure 2A). We optimized the plasticity rule’s parameters (Table S1, set 1) to match the experimental data (Sjöström and Häusser, 2006, Sjöström et al., 2001). At proximal locations (90 μm from the soma; Figure 2B, left panel), a pre-post timing at a frequency of 0.1 Hz led to no change in weight, whereas at and above 10 Hz, in accordance with experiments (Sjöström et al., 2001), LTP was induced. In the model, 0.1-Hz bursts were unable to cause any relevant summation of postsynaptic traces in either LTP pathway. In contrast, at 10 Hz and above, such summation was achieved, leading to LTP. A post-pre timing caused LTD below a frequency of about 30 Hz and LTP beyond 30 Hz both in experiments (Sjöström et al., 2001) and in the model. Here, mainly pre-LTD was initiated in the model at lower frequencies. However, at higher frequencies, summation in both LTP pathways caused the overall switch. When pre-LTD was blocked in the model, this caused even stronger LTP, whereas blockade of pre-LTP substantially reduced the amount of LTP (Figure S2), which is in line with experimental studies (Sjöström et al., 2007). At distal locations (669 μm from the soma; Figure 2B, right panel), LTP was absent for both timings and across all frequencies, whereas frequencies above 20 Hz resulted in slight LTD. Except for pre-post at 50 Hz (Sjöström and Häusser, 2006), no further experimental data were available so all other conditions can be regarded as predictions by the model. Due to the strong attenuation of bAPs (Figure 2A), the local voltage signal at this distance was not strong enough to drive LTP pathways and rather caused LTD.

To illustrate the overall interactions between spike timing, frequency, and location, we created spatiotemporal plasticity windows for this protocol (Figure 2C). At 10 Hz, the plasticity curve at the shortest distance (about 30 μm from the soma) resembled the typical relationship described by classical STDP paradigms (Bi and Poo, 1998). There were local maxima of LTD and LTP close to 0-ms timing, and with increasing delays, the amount of plasticity exponentially decayed to zero in both directions. With increasing distance to the soma, the LTD window (post-pre) became wider, whereas the LTP window (pre-post) rapidly became smaller, as reported in experiments on layer 2/3 (L2/3) pyramidal cells (Froemke et al., 2005) and ultimately vanished at far distal locations. Post–pre timings led to LTD across a wide range of distances from the soma, whereas pre-post timings led to LTP only below 200 μm, which is in accordance with experimental results (Sjöström and Häusser, 2006). At higher frequencies, local peaks were visible in addition to the one at 0 ms. These peaks repeated at multiples of the period (the inverse of the intra-burst frequency). For example, the 20-Hz condition exhibited peaks at −50 ms, 0 ms, and +50 ms. Furthermore, at 40 Hz and 50 Hz, pre-post timings showed a tendency toward overlapping LTP phases, overwriting proximal LTD entirely. This result was partly due to the fact that the presynaptic signals Z and G (which could loosely represent an unknown presynaptic signal and glutamate activation of NMDARs, respectively) decayed slowly and summated at higher frequencies (see Figure 1C). These windows clearly demonstrate that no configuration in this stimulation protocol could evoke distal LTP based solely on the backpropagation of axo-somatic APs, as seen in experiments (Sjöström and Häusser, 2006).

### Burst-Timing-Dependent Plasticity

**Figure 3:**
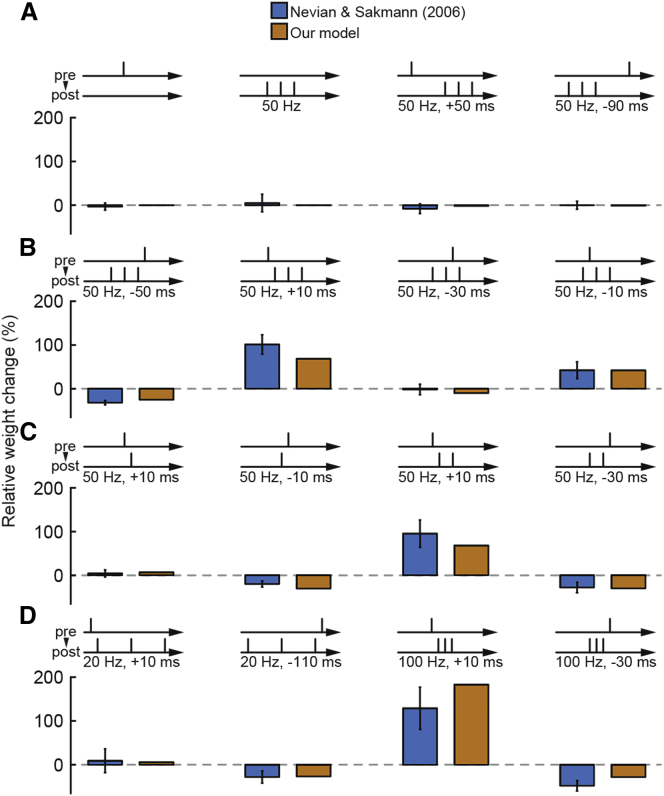
Burst-Timing-Dependent Plasticity (A) STDP induction protocols pairing single presynaptic events with postsynaptic bursts. Comparison between experimental data (blue, mean ± SEM; from Nevian and Sakmann, 2006) and our simulations (orange) for only pre- or postsynaptic activity and for long delays at 50 Hz. Spike timing is defined as the interval between the onset of the presynaptic event and the first step current injection of the postsynaptic burst. (B) As in (A) but with shorter delays between pre- and postsynaptic activity at 50 Hz. (C) As in (A) but with either one or two instead of three postsynaptic events at 50 Hz. (D) As in (A) but with three postsynaptic events at either 20 Hz or 100 Hz.

In a second experimental study, the dynamics of burst-timing-dependent plasticity were studied in basal dendrites of L2/3 pyramidal cells (Nevian and Sakmann, 2006). In this case, one presynaptic spike was paired with a burst consisting of one to three postsynaptic spikes at different frequencies. We implemented the corresponding protocols in a proximal basal dendrite (55 μm from the soma) of the L5 pyramidal neuron model, assuming that the electrophysiological properties of basal dendrites in L2/3 and L5 cells did not differ fundamentally with respect to this stimulation protocol. Using our plasticity rule with adjusted parameters (Table S1, set 2), we showed that all experimental outcomes were reproduced (Figure 3). Plasticity was not induced when there was only pre- or postsynaptic activity or when the delays were too long (Figure 3A). Pure post-pre and pre-post burst pairings at 50 Hz led to similar amounts of LTD and LTP, respectively, whereas intermediate timings caused intermediate effects (Figure 3B). In our model, this was explained by fast saturation of T (which could be loosely linked to postsynaptic VGCC-Ca^2+^; see Figure 1C). Pairings with single postsynaptic events at 50 Hz led to either LTD (−10 ms) or weak LTP (+10 ms), and pairings with two postsynaptic events caused similar results as with three events (Figure 3C). At 20 Hz, pairing one presynaptic spike with three postsynaptic spikes resulted in similar outcomes compared to evoking only one postsynaptic spike at 50 Hz (Figure 3D) because most time constants in the model were too small to cause substantial summation effects. When three postsynaptic spikes were generated at a frequency of 100 Hz, a pre-post timing led to very strong LTP, whereas a post-pre timing led to LTD (Figure 3D). Our plasticity model, thus, faithfully captured all of the outcomes observed in the experimental study. The plasticity changes induced through this set of stimulation protocols were mainly due to pre-LTD and post-LTP in our plasticity rule. Considering the loose biophysical analogies of the pathways, this matched experimental results after pharmacological manipulation, which indicated that LTD depended on activation of mGluRs and Ca^2+^ influx through VGCCs, whereas LTP depended on Ca^2+^ influx through postsynaptic NMDARs (Nevian and Sakmann, 2006).

### Rapid Bursts and Dendritic Ca^2+^ Spikes

**Figure 4:**
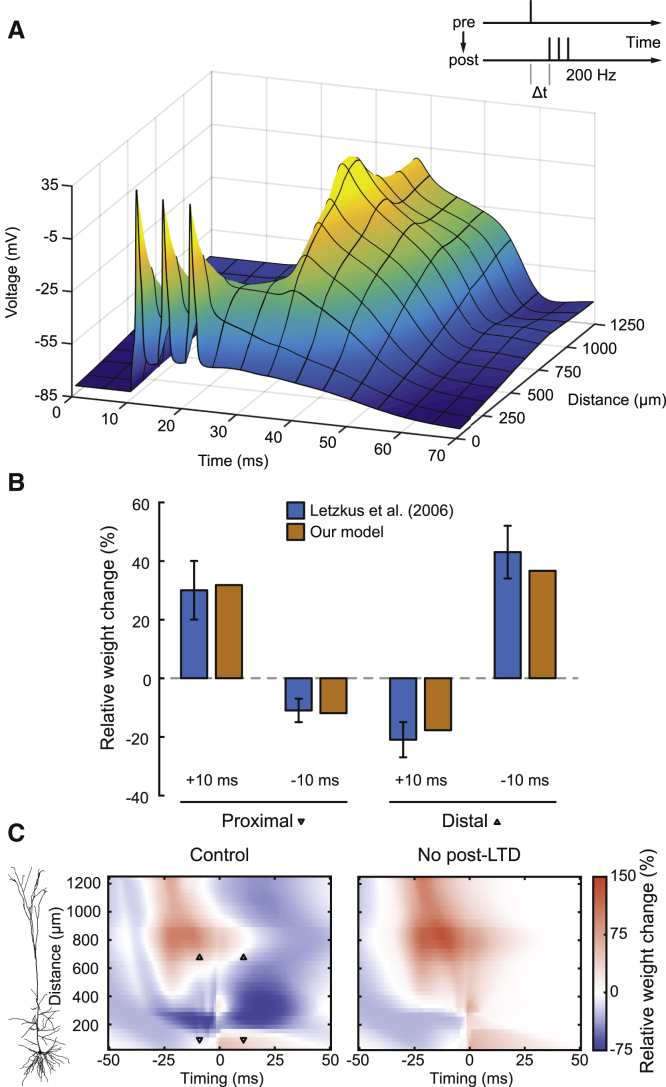
Rapid Bursts and Dendritic Ca^2+^ Spikes (A) Stimulation protocol (Letzkus et al., 2006) (inset) and postsynaptic voltage profile of the neuron model with a dendritic spike induced by high-frequency somatic stimulation (200 Hz) starting at time 10 ms. (B) Relative weight changes in the model (orange) and experimental data (blue, mean ± SEM; from Letzkus et al., 2006, their Figure 5) are plotted as a function of burst timing for proximal (left, 90 μm) and distal (right, 669 μm) locations along the apical dendrite. (C) Spatiotemporal plasticity windows showing relative weight change (color coded) as a function of burst timing (x axis) and distance from the soma (y axis; see cell morphology on the left). In the control condition (left), all pathways were functional. Downward triangles indicate proximal locations, and upward triangles indicate distal locations used for the model simulations in (B). In the blockade condition (right), post-LTD was deactivated. See also Figure S3.

Next, we tested whether active properties of dendrites combined with our plasticity rule could reproduce plastic changes measured at distal synapses where a pre-post-post-post pairing led to LTD and a post-post-post-pre pairing induced LTP (Letzkus et al., 2006). Here, single presynaptic spikes were paired with a rapid (200 Hz) burst of three postsynaptic spikes (Figure 4A, inset). Such rapid bursts were found to sum up distally and evoke dendritic Ca^2+^ spikes, which was reproduced by the L5 pyramidal cell model (Hay et al., 2011) (Figure 4A). We then found a set of parameters (Table S1, set 3) for the plasticity model where simulation results matched the experimental outcomes of all four combinations of timings (+10/−10 ms) and locations (proximal at 90 μm from the soma and distal at 669 μm from the soma). At proximal locations, results of both the experiments and our simulations matched classical STDP, as pre-post led to LTP and post-pre led to LTD (Figure 4B, left). Without any distance-dependent changes to the plasticity rule, our simulations then also captured the experimentally observed plasticity switch at distal locations, where pre-post timings caused LTD and post-pre timings caused LTP (Figure 4B, right). We found the crucial property of these results to be the dendritic spike. It was delayed by about 20 ms compared to the first somatic AP and provided a long-lasting depolarization (Figure 4A). In the model, with pre-post stimulation, the presynaptic signal G at the distal synapse had already decayed substantially when the dendritic spike occurred and, thus, caused only intermediate elevation of C (which could be an abstraction of NMDAR-gated Ca^2+^) over most of the duration (Figure S3, left). Consequently, P was activated more strongly than K (loosely describing phosphatase-kinase competition), which caused post-LTD to surpass post-LTP. Conversely, with post-pre stimulation, the presynaptic event, although 10 ms late, strongly coincided with the delayed dendritic spike, elevating C beyond the threshold $\theta_{C}^{+}$ for a long duration and, thus, causing post-LTP to surpass post-LTD (Figure S3, right). To investigate this in detail, we also visualized the spatiotemporal plasticity window for this induction protocol (Figure 4C, left panel). There was a switch at around 200 μm from the soma where the classical proximal timing requirements changed to more complex distal ones that were shaped by the characteristic voltage curve of the dendritic Ca^2+^ spike. Interestingly, we found that between 200 μm and 400 μm, even the combined depolarization of bAPs and the forward-propagating dendritic spike were below LTP requirements so that only LTD was induced. The four conditions of the experiment (Figures 4C and 4D, triangles) matched the distinct areas in the spatiotemporal plot. We removed post-LTD to illustrate its contribution to these effects, which loosely corresponded to pharmacological inhibition of phosphatases or any other crucial component within this pathway (Figure 4C, right panel). The simulations predict that LTD is then entirely abolished at positive timings, leading to larger LTP areas, whereas pre-LTD remains present at negative timings. We conclude that our plasticity rule, when implemented at synapses on biophysically realistic dendrites with local dendritic Ca^2+^ electrogenesis, is able to reproduce the counterintuitive STDP data for distal synapses (Letzkus et al., 2006).

### Subthreshold Activation of Small Synaptic Clusters

**Figure 5:**
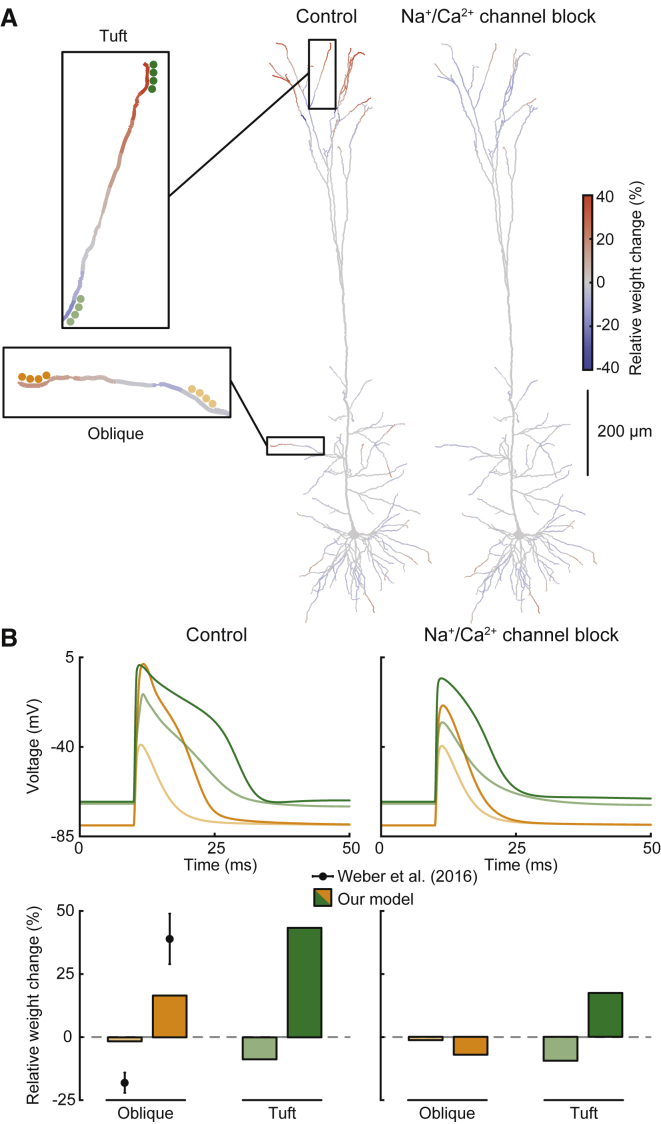
Subthreshold Activation of Small Synaptic Clusters (A) Color-coded dendritic maps of plasticity during subthreshold activation of a cluster of four synapses. Simulations were repeated for all possible locations in the neuron model (boxes on the left show zoomed-in view of example cluster locations). Dendritic maps show average cluster weight changes for control conditions (center) and during block of dendritic Na^+^ and Ca^2+^ channels (right). (B) Voltage traces and average relative weight changes for the example locations shown in the boxes in (A) for both control (left) and channel block (right) conditions. Plasticity data recreated from Weber et al., 2016 Figure 7f, shown as mean ± SEM).

A recent study found that subthreshold activation of a small cluster of four synapses led to plasticity at thin dendritic branches that depended on the relative location of the cluster on the respective branch (Weber et al., 2016). Due to massive increases in input resistance, such thin, distal dendrites are expected to experience considerably more powerful voltage transients caused by synaptic inputs than proximal dendrites (Williams and Stuart, 2002), which could explain these results. We tested this idea by implementing a stimulation protocol in which four synapses located on the same branch segment were strongly activated in rapid succession and repeated simulations for every single segment of the neuron model. Such activation of a small cluster of synapses did indeed result in plasticity, revealing a gradient in relation to the dendritic location (Figure 5A, boxes and center). At proximal, thick dendritic segments, LTD was predominant, which switched to LTP at more distal locations close to the dendritic tips. This gradient was due to dramatic differences in local synaptic potentials that occurred even within single branches (Figure 5B, top left panel). The experimental study focused mainly on oblique dendrites of hippocampal CA1 pyramidal cells (Weber et al., 2016). In our configuration, we found weaker relative weight changes in oblique dendrites of the L5 pyramidal cell model, although our results in distal tuft dendrites agree with the experimental data (Figure 5B, bottom left panel). Generally, at branch points (light orange and green example locations in Figure 5), no plasticity or LTD was common, whereas LTP was typically induced close to the tips (dark orange and green example locations in Figure 5). Due to the absence of a clear post-pre timing in this protocol, pre-LTD was ineffective and the majority of changes were caused by post-LTD and both LTP pathways. We noticed that synaptic potentials in the cell model were boosted by dendritic voltage-dependent Na^+^ and Ca^2+^ channels. Blocking both of these channel types resulted in overall less LTP and more LTD close to the dendritic tips in our specific case (Figures 5A and 5B, right). However, the experimental study showed no significant change in plasticity at oblique dendrites after blocking voltage-dependent Na^+^ channels alone (Weber et al., 2016). We conclude that based on strong input resistance increases, it is possible in thin dendrites to induce bidirectional location-dependent plasticity with subthreshold synaptic inputs alone, as recently observed in some experiments (Sandler et al., 2016, Weber et al., 2016).

### Coincident Activation of Basal and Apical Tuft Inputs

**Figure 6:**
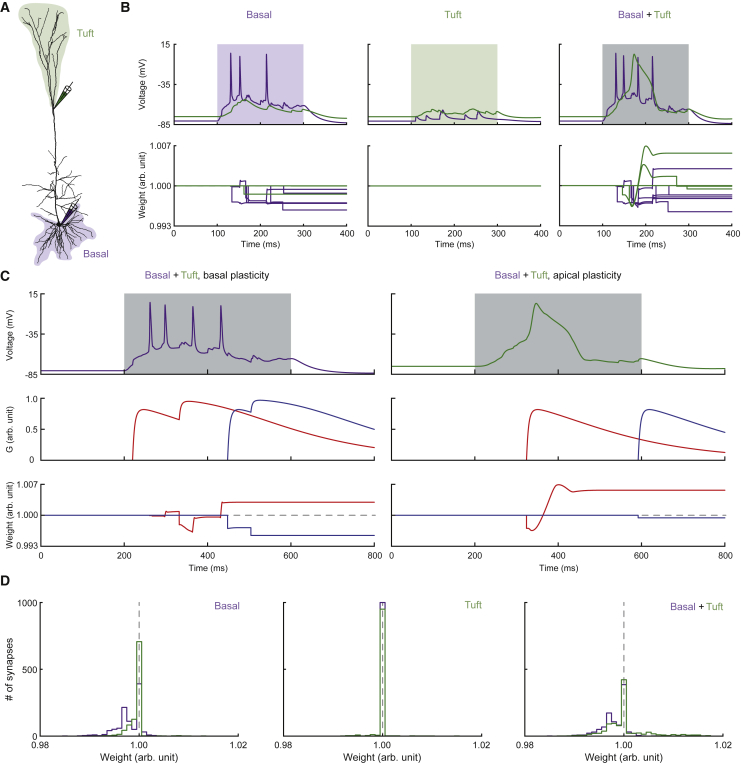
Coincident Activation of Basal and Apical Tuft Inputs (A) Morphology of the neuron model highlighting locations of random background inputs at basal (light purple) and tuft (light green) dendrites, as well as locations of synapses equipped with the plasticity rule at basal (purple pipette) and tuft (green pipette) dendrites. (B) Example voltage traces and weight changes experienced by plastic synapses at basal (purple) and tuft (green) dendrites. Shaded areas indicate intervals of active background inputs, which were either basal alone (left), tuft alone (center), or both (right). (C) Example comparison of most potentiated (red) and most depressed (blue) synapses during coincident basal and tuft inputs, including voltage traces (from B; top right), presynaptic activation G and weight changes over time. (D) Weight distribution histograms of 100 randomly initialized simulations for all three conditions.

After confirming the functionality of our plasticity model by reproducing experimental data of several different stimulation protocols, we predicted how synapses in different locations change in response to more naturally occurring input patterns to pyramidal cells, for which no experimental data exist as of now. As L5 pyramidal cells possess the exclusive property of spanning all six cortical layers, they could potentially act as integrating units for different streams of information (Larkum, 2013). A possible integration mechanism, called backpropagation-activated Ca^2+^ (BAC) firing, involves coincidence of strong proximal and distal inputs that may lead to dendritic spikes and bursts of axo-somatic APs, thereby changing the output mode of the neuron (Larkum et al., 1999), which we expected to have an effect on synaptic plasticity. We implemented a scenario where synapses were placed randomly across basal and tuft regions of the L5b neuron model in a similar way as done in a recent study (Shai et al., 2015) (Figure 6A, shaded areas). In addition to these “background” input synapses, we placed a subset of 10 synapses close to each of the two main spiking zones (Figure 6A, pipette symbols) and equipped them with our plasticity rule. The stimulation protocol consisted of a 100-ms phase of random synaptic activity. In one example, basal activity alone (Figure 6B, left) evoked a few irregularly occurring axo-somatic APs with weak impact on tuft dendrites. Apical synapses did not show plasticity under these circumstances, whereas basal synapses showed a tendency toward LTD. Apical activity alone (Figure 6B, center) did not cause any plasticity in either group of synapses. Coincident activation of both basal and apical synapses (Figure 6B, right) caused BAC firing in the neuron model, evoking both dendritic spikes as well as bursts of APs. There was no absolute switch toward either LTP or LTD in any of the two groups of synapses. Instead, synaptic weights diverged from baseline in both directions. What determines whether a synapse potentiates or depresses during BAC firing? We monitored the time course of synaptic weight during the example simulation for both the most potentiated and most depressed synapse of each group (Figure 6C). This revealed that synapses underwent LTP if they were active during BAC firing but experienced LTD if they were active slightly before or after BAC firing. To assess the underlying weight distributions, we ran 100 simulations with different random seeds, resulting in a total of 1,000 plastic apical and basal synapses in each condition (Figure 6D). We noticed that dendritic spikes were much more common when both groups were concurrently active (probability of about 10% in apical only, 15% in basal only, and 95% in apical and basal conditions). The plasticity results showed that basal activity alone shifted basal synaptic weights toward the LTD regime (Figure 6D, left), whereas apical activity alone did not cause much plasticity (Figure 6D, center). Coincident basal and apical activity led to almost the same distribution of basal weights but opened up LTP for apical synapses (Figure 6D, right). Thus, our simulations predict that BAC firing potentially gates profound bidirectional changes in synaptic weights, especially at apical locations.

### Local Heterosynaptic Effects and NMDA Spikes

**Figure 7:**
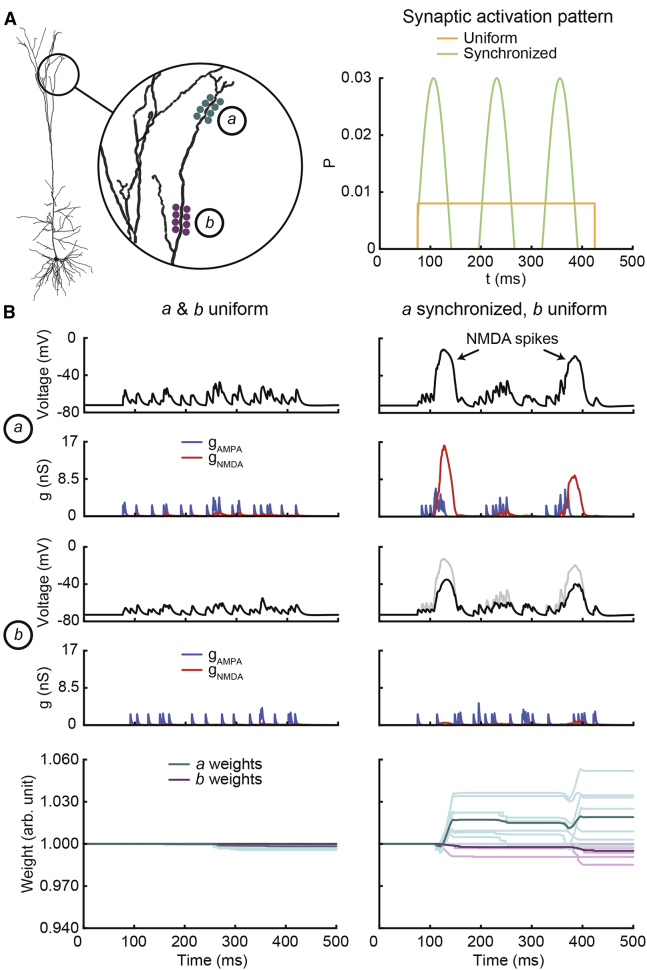
Local Heterosynaptic Effects and NMDA Spikes (A) Morphology of the cell model with zoomed-in view of synaptic cluster locations (left; blue and purple) and probability functions of synaptic events (right), either uniform (orange) or synchronized (green). Probability is given as the chance of a single synapse to activate per step of 1 ms. (B) Simulation results of uniform activity within both clusters (left column) and when cluster *a* was switched to synchronized activity (right column). Traces show local voltage (black, with voltage trace of cluster *a* shown in gray for reference in the panel of cluster *b* in right column), as well as AMPA (blue), and NMDA (red) conductance values summed over all synapses in clusters and weight values of all synapses (light blue and purple lines; dark lines represent averages). Color code as according to (A).

Finally, we wanted to know what our model’s predictions would be regarding heterosynaptic plasticity effects based on local voltage differences. In principle, strong depolarization at one point in the dendrite, e.g., by high-frequency synaptic input, should have the potential to lead to depression in neighboring synapses with low-frequency activity (Jedlicka et al., 2015, Jungenitz et al., 2018) if these synapses experienced depolarization below LTP requirements due to attenuation. We tested this by placing two clusters of 8 synapses each to a thin apical tuft dendrite in the neuron model (location *a*: 1,077 μm from the soma, location *b*: 950 μm from the soma; Figure 7A, left). Synapses in each cluster were then randomly activated in two modes, either uniform (mimicking 8-Hz spontaneous Poisson activity) or synchronized (mimicking 8-Hz stochastic oscillatory activity; Figure 7A, right). Using this protocol, we found that when both clusters were uniformly activated for 350 ms, levels of depolarization were moderate (below −40 mV), NMDA conductances were relatively small, and weights barely changed (Figure 7B, left column). However, when distal synapses at location *a* were switched to synchronized activation, NMDA spikes were elicited, causing strong local depolarization (up to about −12 mV) by substantial increases in NMDA conductance and leading to LTP on average (Figure 7B, right column). In contrast, proximal synapses at location *b* then experienced moderate prolonged depolarization (up to about −35 mV) without major increases in NMDA conductance, resulting mainly in LTD (Figure 7B, right column). Here, the distance of synapses at location *b* from the origin of NMDA spikes was far enough so that depolarization had already been attenuated considerably, preventing LTP and, thus, leading to LTD. These simulations show that local voltage-based heterosynaptic effects can be modeled using our plasticity rule. The results suggest that NMDA spikes could serve as powerful triggers for LTP, but due to their spatially restricted profile might cause opposing effects in weakly active neighboring synapses.

## Discussion

In this study, we developed a synaptic plasticity rule that accounts for a wide range of diverse plasticity experiments and reconciles rate-, timing-, and location-dependent plasticity results with classical Ca^2+^-level-based rules. Our model has two major advantages compared to previous plasticity models. First, whereas most previous rules were developed for point neurons and neglected dendrite morphology, our rule was implemented in a realistic dendritic tree, revealing insights into the interaction between local dynamics of dendritic voltage and plasticity mechanisms. Second, it allows for reproduction of experimental results regarding the dendritic spike-induced switch of LTD/LTP windows at distal apical dendrites of cortical pyramidal cells by using the same set of mechanisms that produce classical STDP at proximal dendrites. In contrast to more traditional plasticity rules that specifically rely on spike timing, our approach accounts for spike timing, frequency, and dendritic location dependence of plasticity induction by computing local signals at the synapse and provides loose analogies to underlying biophysical mechanisms and pathways. In addition to stimulation protocols that involved exclusively axo-somatic APs (Nevian and Sakmann, 2006, Sjöström and Häusser, 2006, Sjöström et al., 2001), our plasticity rule also reproduced results of protocols that led to more complex voltage curves (Letzkus et al., 2006) and subthreshold activation (Weber et al., 2016), strengthening the concept of a more general system of plasticity where STDP is only one emergent property of many (Feldman, 2012, Shouval et al., 2010).

The distance-dependent switch of LTP into LTD in AP-based protocols (Sjöström and Häusser, 2006) (Figure 2) can be explained by voltage attenuation of bAPs, which in our simulations were not powerful enough to activate LTP pathways distally. In contrast, in the protocol with burst-induced dendritic spikes (Letzkus et al., 2006) (Figure 4), several factors contributed to the unique distance-dependent switch of timing requirements. Proximal results in our model were dominated mainly by activation of pre-LTD, pre-LTP, and post-LTP, which have been proposed to be the main pathways involved in STDP protocols (Sjöström et al., 2007). Distally, due to the specific delay of the burst-evoked dendritic spike and the prolonged depolarization it produced, post-LTD became much more prominent, being the only pathway that could cause a decrease in synaptic weight for the pre-post timing (i.e., presynaptic spike preceding postsynaptic burst; Figure 4B; Figure S3). In this specific case, the unique property lay in the combination of a delayed pre-post timing (which enables post-LTD instead of post-LTP due to decay of the presynaptic trace G) with prolonged depolarization (giving post-LTD enough time to have an effect despite its low amplitude). We conclude that intervals that are proximally designated as pre-post and post-pre in this protocol convert to “more delayed pre-post” and “less delayed pre-post” at distal locations, respectively. As such, we think that this behavior represents a mismatch between proximal and distal definitions of spike timing. We further conclude that dendritic-spike-based plasticity is indeed timing dependent but uses a different plasticity window that reflects the different temporal properties of dendritic versus axo-somatic spikes (Figure 4C). All of this was achieved without any distance-dependent mechanistic changes to the rule, suggesting that active dendritic processes are the main determinants of plasticity. At thin, far distal dendrites with high input resistance, activation of small synaptic clusters could cause depolarizing events strong enough to induce plasticity even without local spikes (Weber et al., 2016) (Figure 5). This could also be a possible explanation for the subthreshold plasticity recently found in apical tuft dendrites of L5 pyramidal cells (Sandler et al., 2016). In addition, our results show that the degree to which synaptic cooperativity at a subthreshold level leads to plasticity strongly depends on local dendritic excitability that varies with location. They further indicate that active dendritic properties in the form of voltage-dependent Na^+^ and Ca^2+^ channels could potentially serve to boost these cooperativity-mediated signals, possibly strengthening the plasticity gradient. The demonstration of subthreshold cooperative plasticity is in accordance with modern dendrite-centered theories of memory (Kastellakis et al., 2015, Kastellakis et al., 2016, Legenstein and Maass, 2011), and this opens up the question of how sub- and suprathreshold plasticity signals interact in dendrites.

Our simulations of randomly activated basal and tuft inputs (Figure 6) predict that BAC firing could act as a gateway mechanism for considerable plasticity at both poles of the neuron by generating both bursts of APs and dendritic spikes. They further suggest that only synapses actively contributing to the initiation of BAC firing undergo LTP, whereas those active during other times tend to experience LTD, as expected from a rule with Hebbian character. A possible extension to Hebb’s postulate in this view could be that synapses that cooperate on their quest to associate different inputs potentiate, whereas synapses that do not cooperate and/or do not succeed to establish an associational signal depress. It could, thus, be speculated that BAC firing, although possibly serving as a signal that couples feedforward and feedback information in pyramidal cells (Larkum, 2013), also supports potentiation of those synapses that cause it, thereby increasing the probability that this select subset of synapses leads to BAC firing at the next time they are active. Intriguingly, the plasticity of distal, feedback-associated synapses is a current topic in studies exploring the idea of deep learning principles in the brain (Richards and Lillicrap, 2019).

Finally, we used our plasticity rule to explore heterosynaptic effects within dendritic branches (Figure 7). Notably, we found that NMDA spikes may serve as powerful triggers for LTP. This is biologically plausible because a strong cooperation of synapses is required to elicit NMDA spikes (Major et al., 2013), creating a localized coincidence signal without the need of further synaptic integration. Our results predict that synchronized synaptic activity may cause NMDA spikes and thereby strong LTP (Bono and Clopath, 2017), but this could potentially depress neighboring synapses with uncorrelated activity (Jedlicka et al., 2015, Jungenitz et al., 2018). Although such a process might contribute to synaptic homeostasis (Watt and Desai, 2010), this idea is based purely on dendritic voltage differences in our simulations and currently neglects other mechanisms of heterosynaptic signaling, e.g., by astrocytes (Min et al., 2012) or by competition for resources (Triesch et al., 2018). From the perspective of Ca^2+^-level-based rules, these results further highlight that plasticity gradients may exist not only in time (i.e., via STDP) but also in space by means of localized dendritic potentials.

Our synaptic plasticity rule circumvents two issues that occurred in previous Ca^2+^-amplitude-based models. First, it did not rely on APs with an after-depolarizing tail component to explain LTD at post-pre timings (Shouval et al., 2002), which would be needed to achieve intermediate Ca^2+^ levels in such a condition. In our rule, this interval was covered by pre-LTD, which used low-pass-filtered voltage to detect post-pre timings. Second, a pure amplitude-based model will always exhibit a second LTD window at more delayed pre-post timings (Rubin et al., 2005), which most experimental results do not support. This second LTD window originates from the fact that the coincidence signal has to pass the intermediate zone again every time it decreases, which happens as a consequence of increased pre-post delay. In our plasticity rule, because post-LTD was set to a low amplitude compared to post-LTP in all simulations, it was induced in negligible amounts during brief depolarizations, such as from APs, even at delayed pre-post timings. However, long-lasting depolarizations, such as dendritic spikes, could lead to temporal integration of considerable amounts of post-LTD, as seen in our simulations of the burst-induced dendritic spike protocol. Thus, in addition to the amplitude hypothesis, our rule also relates to the so-called duration hypothesis, which states that long-lasting intermediate Ca^2+^ pulses are needed for (post-)LTD, whereas short-duration, high-amplitude Ca^2+^ pulses induce LTP (Evans and Blackwell, 2015). Another hypothesis that our rule harmonizes well with is that there might be segregated Ca^2+^ pools, which could come in the form of Ca^2+^ micro- or nanodomains (Evans and Blackwell, 2015), originating from distinct sources (Jedlicka and Deller, 2017) and feeding strongly localized messenger chains. Such a system involving multiple coincidence detectors is in accordance with previous theoretical and experimental studies indicating that independent VGCC- and NMDAR-activated pathways exist (Bender et al., 2006, Karmarkar and Buonomano, 2002, Oliet et al., 1997, Padamsey et al., 2017, Pigott and Garthwaite, 2016, Sjöström et al., 2003, Sjöström et al., 2007).

Because our model is based on a previously developed voltage-dependent STDP rule (Clopath and Gerstner, 2010, Clopath et al., 2010), it might be worth highlighting some common key elements. Notably, the implementation of pre-LTD in our plasticity rule was almost identical to LTD in the Clopath et al. (2010) rule, where the combination of a discrete presynaptic event with low-pass-filtered postsynaptic voltage allowed for precise post-pre coincidence detection. There were also similarities between LTP pathways in our plasticity rule and LTP in the Clopath et al. (2010) rule. In both implementations, a slow presynaptic trace was multiplied with multiple factors based on postsynaptic voltage to detect pre-post coincidence. However, in contrast to our model presented here, there is no LTD mechanism in the Clopath et al. (2010) model that is activated at delayed pre-post timings, even during long-lasting depolarizations.

Numerous extensions could be made to our framework to increase precision and flexibility with regard to the broad range of plasticity-induction protocols. As more detailed biophysical models of receptors and proteins emerge, they could replace the phenomenological components in our plasticity framework. This could eventually lead to a realistic, fully biophysical model of plasticity induction. For instance, our presynaptic signal D could be replaced by realistic modeling of mGluR signaling, and our postsynaptic pathway activation variables P and K could be substituted by kinetic models of phosphatases, kinases, and their binding agents. Furthermore, we had to readjust plasticity amplitudes (representing the impact of each plasticity pathway) as parameters to reproduce different experiments (see Table S1; see also Figure S4). These adjustments could reflect differences between these experiments, including methodological details (e.g., animal age, recording temperature, and ionic composition of solutions), differences between synapse types (Larsen and Sjöström, 2015), and the lack of plasticity maintenance mechanisms in our rule. In addition, for simulations of longer time intervals and in networks, concepts such as short-term plasticity (Zucker and Regehr, 2002), neuromodulation (Foncelle et al., 2018, Gerstner et al., 2018), synaptic scaling (Turrigiano, 2008), and metaplasticity (Jedlicka et al., 2015) would be needed.

In summary, our simulations indicate that a single general plasticity rule is sufficient to reproduce different outcomes of plasticity experiments at various dendritic locations, providing a unification of classical STDP and Ca^2+^-level-based rules. Our plasticity rule can be readily combined with detailed neuron models to explore STDP as well as plasticity mediated by dendritic Ca^2+^ and Na^+^ spikes, NMDA spikes, subthreshold activation of synaptic clusters, and any combination of these concepts.

## STAR★Methods

### Key Resources Table

**Table:** 

REAGENT or RESOURCE	SOURCE	IDENTIFIER
**Software and Algorithms**		

Cortical L5b pyramidal cell model	Hay et al., 2011	ModelDB: 139653
Four-pathway phenomenological synaptic plasticity model	This paper	ModelDB: 251493
NEURON	Carnevale and Hines, 2006	RRID: SCR_005393
ModelDB	McDougal et al., 2017	RRID: SCR_007271

### Lead Contact and Materials Availability

Further information and requests for resources should be directed to and will be fulfilled by the Lead Contact, Christian Ebner (ebner@fias.uni-frankfurt.de). This study did not generate new unique reagents.

### Method Details

#### Plasticity Rule

Our plasticity rule quantifies the activation of its four separate pathways (Figures 1B, 1C, and S1) directly from the timing of a presynaptic event and the local postsynaptic membrane voltage.

##### Presynaptic LTD

Presynaptic LTD in this model was inspired by mGluR-CB1R-LTD (Heifets and Castillo, 2009) (Figures 1A and 1B, left panels). For simplification, local postsynaptic membrane potentials u were dimensionless quantities. $\bar{T}$ was then a low-pass filtered version of the portion of u that was above a threshold $\theta_{u}^{T}$ with a time constant $\tau_{T}$

(1) $$\tau_{T}\cdot \frac{d}{dt}\cdot \bar{T}\left(t\right)=-\bar{T}\left(t\right)+{\left[u\left(t\right)-\theta_{u}^{T}\right]}_{+},$$

where for any given value x, the notation ${\left[x\right]}_{+}$ indicated a rectifier, defined as being x for positive values of x and 0 in all other cases. Using $\bar{T}$, we calculated the trace T

(2) $$T\left(t\right)=tanh\left(b_{T}\cdot \bar{T}\left(t\right)\right)$$

(3) $$b_{T}=\frac{\text{ln}\left(m_{T}\right)}{2}.$$

The hyperbolic tangent was used as a sigmoid saturation function in multiple instances below to provide a soft boundary for variables of the model and to loosely relate to binding kinetics of the agents involved. The characteristic saturation in the case of T was determined by the specific slope $m_{T}$ (Table S1) and different slopes according to Equation 3 were used to compute other traces (see below). We further defined the presynaptic variable D as a series of delta pulses

(4) $$D\left(t\right)=\sum_{i}\delta \left(t-t_{i}\right),$$

with $t_{i}$ representing times of presynaptic events. The coincidence of pre- and postsynaptic signals E was therefore given by

(5) E(t)=D(t)⋅T(t),

which was used as a direct indicator of pre-LTD. A possible link to biophysical processes could be the following: The postsynaptic trace T could loosely represent the amount of VGCC-gated Ca^2+^ that was bound to PLC at a given time. A minimal depolarization $\theta_{u}^{T}$ would then be required to open the VGCCs (simplified with a linear increase in permeability), while the binding/unbinding rate of Ca^2+^ from PLC would be determined by $\tau_{T}$. The delta pulses in D could be related to the signaling cascades evoked by mGluRs upon glutamate binding and E could loosely represent the amount of synthesized eCBs by PLC (Hashimotodani et al., 2005).

##### Presynaptic LTP

Presynaptic LTP was inspired by NO-LTP (Padamsey et al., 2017, Pigott and Garthwaite, 2016, Sjöström et al., 2007) (Figures 1A and 1B, left panels). In a similar way as with T, we defined another trace $N_{\alpha }$ based on low-pass filtered postsynaptic voltage

(6) $$\tau_{N}^{\alpha }\cdot \frac{d}{dt}\cdot {\bar{N}}_{\alpha }\left(t\right)=-{\bar{N}}_{\alpha }\left(t\right)+{\left[u\left(t\right)-\theta_{u}^{N}\right]}_{+},$$

(7) $$N_{\alpha }\left(t\right)=tanh\left(b_{N}^{\alpha }\cdot {\bar{N}}_{\alpha }\left(t\right)\right),$$

using a slope $m_{N}^{\alpha }$ (Table S1) via $b_{N}^{\alpha }$ (as according to Equation 3). We then defined a second postsynaptic trace $N_{\beta }$ from

(8) $$\tau_{N}^{\beta }\cdot \frac{d}{dt}\cdot {\bar{N}}_{\beta }\left(t\right)=-{\bar{N}}_{\beta }\left(t\right)+{\bar{N}}_{\alpha }\left(t\right),$$

(9) $$N_{\beta }\left(t\right)=tanh\left(b_{N}^{\beta }\cdot {\bar{N}}_{\beta }\left(t\right)\right),$$

using a slope $m_{N}^{\beta }$ via $b_{N}^{\beta }$. The product of $N_{\alpha }$ and $N_{\beta }$ surpassing a threshold $\theta_{N}$ was defined as N

(10) $$N\left(t\right)={\left[N_{\alpha }\left(t\right)\cdot N_{\beta }\left(t\right)-\theta_{N}\right]}_{+}.$$

In addition, a presynaptic activity trace Z was shaped by the difference of two exponentials and application of the hyperbolic tangent

(11) $$Z\left(t\right)=tanh\left(b_{Z}\cdot \left(Z_{b}\left(t\right)-Z_{a}\left(t\right)\right)\right),$$

(12) $$\tau_{Z}^{a}\cdot \frac{d}{dt}\cdot Z_{a}\left(t\right)=-Z_{a}\left(t\right)+\varepsilon_{Z}\cdot D\left(t\right),$$

(13) $$\tau_{Z}^{b}\cdot \frac{d}{dt}\cdot Z_{b}\left(t\right)=-Z_{b}\left(t\right)+\varepsilon_{Z}\cdot D\left(t\right),$$

(14) $$\varepsilon_{Z}=\frac{1}{-e^{-\frac{\omega_{Z}}{\tau_{Z}^{a}}}+\;e^{-\frac{\omega_{Z}}{\tau_{Z}^{b}}}},$$

(15) $$\omega_{Z}=\frac{\tau_{Z}^{a}\cdot \tau_{Z}^{b}}{\tau_{Z}^{b}-\tau_{Z}^{a}}\cdot \text{ln}\left(\frac{\tau_{Z}^{b}}{\tau_{Z}^{a}}\right).$$

Here, the triggering of a presynaptic event via the event times in D (Equation 4) also elevated Z in a time course characterized by the time constants $\tau_{Z}^{a}$ and $\tau_{Z}^{b}$. The sole purpose of $\varepsilon_{Z}$ was to normalize the peak of the trace to 1. Coincidence of presynaptic signals Z and postsynaptic signals N yielded X

(16) X(t)=Z(t)⋅N(t),

which was used as the indicator for pre-LTP. A possible link of our implementation of pre-LTP to biophysical mechanisms could be a recently described presynaptic form of LTP (Padamsey and Emptage, 2013). In this view, $N_{\alpha }$ could be related to the influx of Ca^2+^ via L-VGCCs (Pigott and Garthwaite, 2016) with a relatively high voltage threshold $\theta_{u}^{N}$ (see Table S1), while $N_{\beta }$ could be related to a slower process based on $N_{\alpha }$ such as CaM binding. Based on this perspective, N could be a loose analogy to NOS activation and NO synthesis via CaM (Abu-Soud et al., 1994), while X could refer to a yet unknown presynaptic coincidence detector based on a presynaptic signal Z (Padamsey et al., 2017).

##### Postsynaptic LTD

Postsynaptic LTD in our model was loosely based on NMDAR-LTD (Lüscher and Malenka, 2012) (Figures 1A and 1B, right panels). Here, a third presynaptic trace G was shaped by the difference of two exponential functions in the same way as Z ((11), (12), (13), (14), (15)), but with time constants $\tau_{G}^{a}$ and $\tau_{G}^{b}$ and a saturation slope $m_{G}$. We then computed C as the coincidence of G and u above $\theta_{u}^{C}$

(17) $$C\left(t\right)=G\left(t\right)\cdot {\left[u\left(t\right)-\theta_{u}^{C}\right]}_{+}.$$

Based on C, a trace P was calculated as

(18) $$P\left(t\right)={\left[C\left(t\right)-\theta_{C}^{-}\right]}_{+}\cdot {\left[\theta_{C}^{+}-C\left(t\right)\right]}_{+}\cdot \frac{1}{{\left(\frac{\theta_{C}^{+}-\theta_{C}^{-}}{2}\right)}^{2}}.$$

P was chosen to be a quadratic function of C between the thresholds $\theta_{C}^{-}$ and $\theta_{C}^{+}$. The peak of the quadratic function was normalized to 1 by removing its dependence on $\theta_{C}^{-}$ and $\theta_{C}^{+}$, which we found to be useful for optimizing the plasticity amplitude. The amount of post-LTD was correspondingly directly dependent on P. This implementation of post-LTD could be interpreted as a loose analogy to the following biophysical processes: G could represent the total amount of activated NMDARs following glutamate binding, where binding and unbinding kinetics could be determined by $\tau_{G}^{a}$ and $\tau_{G}^{b}$, respectively. In our implementation, multiple events summed up in G but were limited to a maximum of 1 via the saturating process. This could correspond to the existing proposal that NMDARs of a synapse are not fully saturated upon a single release event (Ishikawa et al., 2002, Mainen et al., 1999). In this view, C could loosely represent the total fraction of open NMDARs, where $\theta_{u}^{C}$ could be related to the minimal voltage required to release the Mg^2+^ block. The threshold $\theta_{C}^{-}$ could mark the minimal amount of Ca^2+^ needed to activate phosphatases and therefore the start of LTD induction along the Ca^2+^ continuum. The threshold $\theta_{C}^{+}$ on the other hand could then designate the amount of Ca^2+^ where the competition between phosphatases and kinases reaches an equilibrium and therefore would mark the start of LTP along the continuum. The maximum of the function, located at the center between $\theta_{C}^{-}$ and $\theta_{C}^{+}$, could then loosely represent the amount of Ca^2+^ where phosphatases are most active (Lisman, 1989).

##### Postsynaptic LTP

Postsynaptic LTP was inspired by NMDAR-LTP (Lüscher and Malenka, 2012) (Figures 1A and 1B, right panels). In our model, post-LTP depended on the coincidence of three traces, denoted $K_{\alpha }$, $K_{\beta }$ and $K_{\gamma }$. $K_{\alpha }$ from

(19) $$K_{\alpha }\left(t\right)=tanh\left(b_{K}^{\alpha }\cdot {\left[C\left(t\right)-\theta_{C}^{+}\right]}_{+}\right)\cdot \rho \left(t\right),$$

was limited to a maximum of 1 via the hyperbolic tangent using a slope $m_{K}^{\alpha }$ via $b_{K}^{\alpha }$ (Table S1; see Equation 3). $K_{\alpha }$ was additionally limited by a variable ρ

(20) $$\rho \left(t\right)=1-K_{\beta }\left(t\right).$$

Since $K_{\beta }$ was dependent on $K_{\alpha }$ (see below), ρ served as a negative feedback signal, thus ensuring that the sum of $K_{\alpha }$ and $K_{\beta }$ could not be greater than 1. $K_{\beta }$ itself was a low-pass filtered version of $K_{\alpha }$

(21) $$\tau_{K}^{\beta }\cdot \frac{d}{dt}\cdot {\bar{K}}_{\beta }\left(t\right)=-{\bar{K}}_{\beta }\left(t\right)+K_{\alpha }\left(t\right),$$

(22) $$K_{\beta }\left(t\right)=tanh\left(b_{K}^{\beta }\cdot s_{K}^{\beta }\cdot {\bar{K}}_{\beta }\left(t\right)\right),$$

where $s_{K}^{\beta }$ was a factor simply used to scale up ${\bar{K}}_{\beta }$ into the range of the saturation function with slope $m_{K}^{\beta }$ via $b_{K}^{\beta }$. It was then further low-pass filtered with a time constant $\tau_{K}^{\gamma }$ to compute $K_{\gamma }$

(23) $$\tau_{K}^{\gamma }\cdot \frac{d}{dt}\cdot K_{\gamma }\left(t\right)=-K_{\gamma }\left(t\right)+K_{\beta }\left(t\right).$$

K was then the product of all three traces

(24) $$K\left(t\right)=K_{\alpha }\left(t\right)\cdot K_{\beta }\left(t\right)\cdot K_{\gamma }\left(t\right),$$

which was directly used as the indicator of post-LTP. A loose analogy to biophysical processes could be the following: $K_{\alpha }$ could be related to the instantaneous activation of CaMKII by Ca^2+^-CaM after enough Ca^2+^ passed NMDARs so that kinase activation surpassed phosphatase activation, illustrated by the amount of C above $\theta_{C}^{+}$ (Lisman, 1989). In this view, the negative feedback trace ρ could represent competition among proteins in different states regarding the limited amounts of free CaM in dendritic spines (Persechini and Stemmer, 2002). $K_{\beta }$ could then be related to a slower process, such as the amount of a certain configuration of Ca^2+^-CaM bound to CaMKII (Pepke et al., 2010) or possibly trapped (Meyer et al., 1992), decaying with a time constant $\tau_{\beta }$. $K_{\gamma }$ could be linked to a process based on $K_{\beta }$, such as another configuration of Ca^2+^-CaM with even slower kinetics (Pepke et al., 2010) or a slow conformational change which might be required for autophosphorylation of CaMKII (Chao et al., 2010). Finally, K could loosely illustrate the amount of CaMKII that reaches the autonomous state and/or binds to NR2B subunits at any given time, which both have been proposed to be crucial for LTP (Lisman et al., 2012).

##### Synaptic Weight

Synaptic weight was the product of both pre- and postsynaptic weight factors

(25) $$w\left(t\right)=w_{pre}\left(t\right)\cdot w_{post}\left(t\right).$$

The factors were each updated by the sum of their respective pathway indicators

(26) $$\frac{d}{dt}\cdot w_{pre}\left(t\right)=-A_{pre}^{LTD}\cdot E\left(t\right)+A_{pre}^{LTP}\cdot X\left(t\right)\cdot \eta ,$$

(27) $$\frac{d}{dt}\cdot w_{post}\left(t\right)=-A_{post}^{LTD}\cdot P\left(t\right)\cdot \eta +A_{post}^{LTP}\cdot K\left(t\right)\cdot \eta ,$$

where $A_{pre}^{LTD}$, $A_{pre}^{LTP}$, $A_{post}^{LTD}$ and $A_{post}^{LTP}$ were the respective pathway amplitudes (Table S1). Due to its calculation via delta pulses, the pre-LTD pathway was inherently invariant to changes in integration step size. In contrast, the other three pathways were integrated over time and continuously (i.e., at each step) updated. We thus introduced a learning rate η ($\eta =0.025\;{\mathrm{ms}}^{-1}$ for all of our simulations) to make them independent of changes in integration step size. The presynaptic weight $w_{pre}$ could be related to transmitter release probability, although our model did not explicitly calculate probabilities and synaptic responses should all be regarded as averages. We thus set the hard bounds to $0\leq w_{pre}\leq 1$. The postsynaptic weight $w_{post}$, which would be interpreted as a factor contributing to postsynaptic current, was limited via $0\leq w_{post}\leq 5$. These bounds prevented both the occurrence of negative weights and excessively strong synapses. In the beginning of each simulation, weight factors were initialized to $w_{pre}^{init}=0.5$ and $w_{post}^{init}=2$, leading to a total weight $w^{init}=1$.

#### Synaptic currents

Synaptic currents were computed as sums of both AMPAR- and NMDAR-mediated components. The AMPAR component was calculated via

(28) $$g_{AMPA}\left(t\right)=s_{AMPA}\cdot w_{post}\left(t\right)\cdot \left(g_{AMPA}^{b}\left(t\right)-g_{AMPA}^{a}\left(t\right)\right),$$

(29) $$\tau_{AMPA}^{a}\cdot \frac{d}{dt}\cdot g_{AMPA}^{a}\left(t\right)=-g_{AMPA}^{a}\left(t\right)+\varepsilon_{AMPA}\cdot D\left(t\right)\cdot g_{max},$$

(30) $$\tau_{AMPA}^{b}\cdot \frac{d}{dt}\cdot g_{AMPA}^{b}\left(t\right)=-g_{AMPA}^{b}\left(t\right)+\varepsilon_{AMPA}\cdot D\left(t\right)\cdot g_{max},$$

where D indicated event times (see Equation 4). The time constants describing glutamate kinetics were $\tau_{AMPA}^{a}=0.2\;\mathrm{ms}$ and $\tau_{AMPA}^{b}=2\;\mathrm{ms}$ and $\varepsilon_{AMPA}$ was calculated from these time constants according to ((14), (15)). $g_{max}$ was the maximum synaptic conductance, which in addition to NMDA/AMPA ratio constants $s_{AMPA}$ and $s_{NMDA}$ was set individually for each stimulation protocol (see further below). The NMDAR component was calculated via

(31) $$g_{NMDA}\left(t\right)=s_{NMDA}\cdot w_{post}^{init}\cdot G\left(t\right)\cdot g_{max}\cdot \frac{1}{1+e^{-0.08\cdot u\left(t\right)}\cdot 3.57^{-1}},$$

where glutamate kinetics were modeled via the difference of exponentials in G, using $\tau_{G}^{a}=2\;\mathrm{ms}$ and $\tau_{G}^{b}=50\;\mathrm{ms}$ (Poleg-Polsky, 2015) (Table S1) and the last factor described the Mg^2+^ block depending on local voltage u (Jahr and Stevens, 1990, Rhodes, 2006). Finally, synaptic currents were computed from

(32) $$g_{syn}\left(t\right)=w_{pre}\left(t\right)\cdot \left(g_{AMPA}\left(t\right)+g_{NMDA}\left(t\right)\right),$$

(33) $$I_{syn}\left(t\right)=g_{syn}\left(t\right)\cdot \left(V\left(t\right)-E_{syn}\right),$$

where V was the local membrane voltage in the cell model and $E_{syn}$ was the reversal potential, which we set to 0 mV.

#### Neuron Model

In our simulations, the plasticity rule was applied to a pyramidal neuron model developed by Hay et al. (2011). It represents a cortical L5b pyramidal cell of the rat whose morphology was reconstructed in 3D via light microscopy. Electrophysiological properties were acquired by current injection protocols combined with whole-cell recording techniques *in vitro* and subsequently reproduced in the cell model using a multi-objective genetic algorithm. Of the four biophysical ion channel configurations provided by the authors, we selected the fourth one (see their supplementary materials, Hay et al., 2011), where the APs are generated in the axon initial segment.

#### Implementation

Simulations were run using the NEURON 7.4 environment (Carnevale and Hines, 2006) using a constant integration time step of 0.025 ms. Presynaptic signals were sent directly to the synapse without explicit modeling of a presynaptic cell. Fitting of the plasticity model parameters was done via manual search in a two-stage process. First, all time constants, thresholds and saturation slopes were adjusted so that all simulations would describe their respective experimental data qualitatively. Then, plasticity pathway amplitudes were fine-tuned for the three stimulation protocols used in experiments, aiming at quantitative matches wherever possible (Table S1). To save simulation time of repetitive stimulation protocols, we ran one sweep at a time and approximated the final outcome via

(34) $$w^{final}=\left(w_{pre}^{init}+\left(w_{pre}^{sweep}-w_{pre}^{init}\right)\cdot n\right)\cdot \left(w_{post}^{init}+\left(w_{post}^{sweep}-w_{post}^{init}\right)\cdot n\right),$$

where $w_{pre}^{sweep}$ and $w_{post}^{sweep}$ were weight factors after one sweep and n was the total number of sweeps in the protocol.

#### Stimulation protocols

##### Voltage Clamp

The membrane potential of the neuron model was clamped to voltages in the range of –75 to –15 mV (Figure 1E). Single presynaptic events were directly sent to the plasticity rule (using parameter set 1; see Table S1) and ten of these sweeps were taken into account to calculate final weight changes.

##### Pre- and Postsynaptic Bursts

The stimulation procedure was implemented according to previous experiments (Sjöström and Häusser, 2006, Sjöström et al., 2001) (Figure 2A). Stimulation was performed by injecting step currents (5 ms at 2.7 nA) into the soma of the cell model to evoke bursts of five axo-somatic APs. Simulations included one pre- and one postsynaptic burst at a time, shifted by either +10 ms or –10 ms. Ten of these sweeps were considered for an intra-burst frequency of 0.1 Hz (representing 50 spikes in total) and 15 sweeps were used for intra-burst frequencies of 10, 20, 40 and 50 Hz (representing 75 spikes in total), matching the experimental procedure (Sjöström et al., 2001). To assess location differences, one proximal (90 μm from soma) and one distal (669 μm from soma) location along the apical dendrite were chosen, at which the plasticity rule (using parameter set 1; see Table S1) was placed. Proximal locations mimicked L5→L5 connections, while distal locations mimicked L2/3→L5 connections (Sjöström and Häusser, 2006). We set $g_{max}=3.5\;\mathrm{nS}$ and $s_{AMPA}=s_{NMDA}=0.5$.

##### Single Presynaptic Events and Postsynaptic Bursts

In this stimulation protocol, several combinations of timings, burst frequencies and numbers of spikes were tested (Nevian and Sakmann, 2006) (Figure 3). To match the experiments, we applied the plasticity rule (using parameter set 2; see Table S1) to a proximal basal dendrite (55 μm from the soma). In our simulations, postsynaptic APs were evoked via somatic step current injection (5 ms at 2.1 nA). Single sweeps were simulated and 60 sweeps used for calculation of weights (corresponding to 60 low-frequency repetitions). Exact configurations of all the different spike patterns are visualized in Figure 3. We set $g_{max}=3.5\;\mathrm{nS}$ and $s_{AMPA}=s_{NMDA}=0.5$.

##### Burst-Induced Dendritic Spikes

Following the corresponding experiments (Letzkus et al., 2006), one presynaptic event was either followed (+10 ms) or preceded (–10 ms) by a burst of three postsynaptic APs at 200 Hz (Figure 4A). APs in our simulations were evoked via somatic step current injection (2 ms at 5.5 nA) to reliably produce distal dendritic Ca^2+^ spikes (see voltage traces in Figures 4A and S3). Proximal and distal locations for the plasticity rule (using parameter set 3; Table S1) in these simulations were 90 μm and 669 μm from the soma, respectively. Single sweeps were simulated and 100 sweeps used for calculations, representing 100 low-frequency repetitions. We set $g_{max}=3.5\;\mathrm{nS}$ and $s_{AMPA}=s_{NMDA}=0.5$.

##### Voltage Profiles and Plasticity Windows

To generate the spatiotemporal voltage plots (Figures 2C and 4C), we selected one termination point of an apical dendrite in the model, calculated the exact path down to the soma and selected locations roughly every 30 μm (limited by the compartmental resolution of the cell model), leading to a set of 41 more or less evenly distributed locations along the path. We then applied the exact current injection protocol of each given experiment and measured voltages at all selected locations over the entire duration. Voltage curves did not change considerably with respect to distance for alternative paths (i.e., where another termination point was chosen). For the spatiotemporal plasticity windows, we used the set of 41 locations along one specific path to apply the plasticity rule to and simulated each stimulation protocol with different timings in an interval of [–50 50] ms at steps of 1 ms. Each data point of the image thus corresponded to one of the resulting 4,141 single simulations.

##### Subthreshold Activation of Synapse Clusters

This stimulation protocol involved activation of a cluster of four synapses in rapid succession (0.1 ms interval), imitating two-photon glutamate uncaging experiments (Weber et al., 2016). We repeated the protocol, each time placing the cluster (using parameter set 3; see Table S1) at a different segment of the neuron model for all possible segments and then mapped plasticity outcomes onto the morphology (Figure 5A). We used 50 sweeps, representing 50 low-frequency repetitions. In the channel block condition, we simply set the conductance of all apical Na^+^ and Ca^2+^ channels of the neuron model to zero. Example locations (Figure 5A, boxes) were chosen to be at 20% and 90% of the total branch length, respectively, in accordance with experiments (Weber et al., 2016). For each of the four synapses, we set $g_{max}=2.5\;\mathrm{nS}$, $s_{AMPA}=0.8$ and $s_{NMDA}=0.2$ to prevent excessive NMDA currents, as these were not reported in the study (Weber et al., 2016).

##### Random Basal and Tuft Inputs

We randomly distributed non-plastic input synapses across parts of the dendritic tree, amounting to 50 basal and 300 tuft synapses with an AMPAR-exclusive conductance of 2.5 nS each. In addition, we placed ten plastic synapses (using parameter set 3; see Table S1) close to each of the two main spiking zones of the cell (basal: 32 μm from the soma; apical: 672 μm from the soma; Figure 6A). A single sweep in the simulations consisted of a phase of synaptic activity (100 ms). During active phases, either basal, apical or all synapses were randomly activated independently using a Poisson distribution at an average frequency of 10 Hz. For the weight distribution histograms (Figure 6D), we ran 100 simulations with different random seeds per condition, leading to a total of 1,000 plastic synapses per location and condition. For each of the plastic synapses, we set $g_{max}=2.5\;\mathrm{nS}$, $s_{AMPA}=0.8$ and $s_{NMDA}=0.2$.

##### Heterosynaptic Effects and NMDA Spikes

This stimulation protocol involved two clusters of eight synapses each, which we placed on a far distal apical tuft dendrite of the cell model (Figure 7A). The distances to the soma were 950 and 1,077 μm, respectively. Synaptic activation patterns were available in two modes, uniform and synchronized. Uniform activation was modeled using a Poisson distribution with an average frequency of 8 Hz. Synchronized activation was modeled using a sinusoid with a frequency of 8 Hz and amplitude of 0.05 oscillating around 0.005, where positive function values gave probabilities of synapses being activated per 1 ms. The protocol consisted of one phase of synaptic activity at both clusters with a duration of 350 ms. For each of the synapses, we set $g_{max}=2.5\;\mathrm{nS}$ and $s_{AMPA}=s_{NMDA}=0.5$.

#### Sensitivity Analysis

We performed sensitivity analysis by varying each single parameter in the model by four different factors for each set of amplitudes (Figure S4). The model is relatively sensitive especially to changes in threshold parameters, which in most extreme cases can lead to pathways being activated even at resting potential.

### Quantification and Statistical Analysis

For experimental results reproduced by our model, original data is always given as mean ± SEM (see Figures 2B, 3, 4B, and 5B). Simulation data presented in the histograms of Figure 6D was acquired using 100 different random seeds for generating Poisson-distributed event sequences in 10 plastic synapses each, leading to n = 1,000 plastic synapses per location and condition. Identical seeds were used between conditions.

### Data and Code Availability

The accession number for the stimulation procedures and the plasticity model reported in this paper is ModelDB: 251493.

## Supplemental Information

Document S1. Figures S1–S4 and Table S1

Document S2. Article plus Supplemental Information
