# Supplementary material for: Unifying Long-Term Plasticity Rules for Excitatory Synapses by Modeling Dendrites of Cortical Pyramidal Neurons
Source: Cell Rep. 2019 Dec 24;29(13):4295–4307.e6. doi: 10.1016/j.celrep.2019.11.068 (PMC6941234; doi:10.1016/j.celrep.2019.11.068)
Supplement: Document S1. Figures S1–S4 and Table S1 [file mmc1.pdf]

Cell Reports, Volume *29*

Supplemental Information

Unifying Long-Term Plasticity Rules for Excitatory Synapses by Modeling Dendrites of Cortical Pyramidal Neurons

Christian Ebner, Claudia Clopath, Peter Jedlicka, and Hermann Cuntz

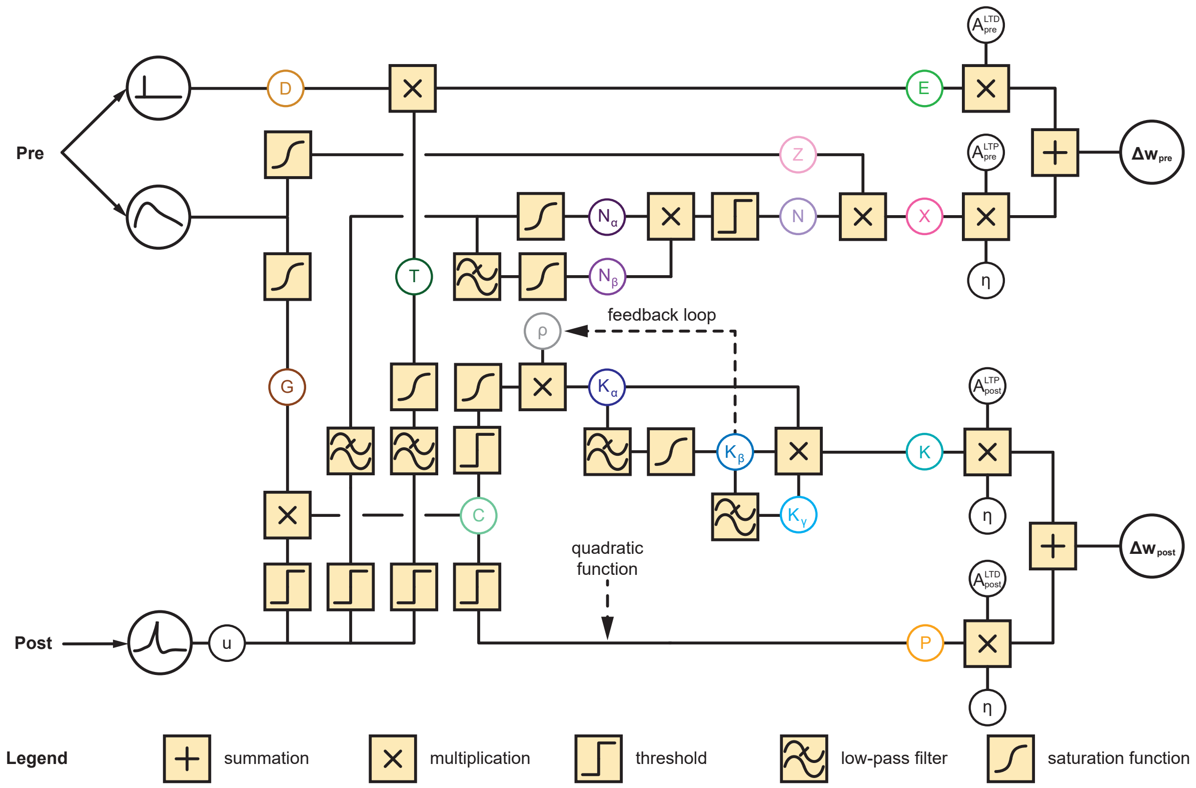

Figure S1. Plasticity model overview. Related to Figure 1 and STAR⋆Methods. Calculations carried out within the model are illustrated in a flow diagram, starting with raw pre and post signals on the left and leading to synaptic weight change on the right. Variables are color-coded as according to Figure 1 in the main text. Results of summations and multiplications are always located on the right of their respective symbols.

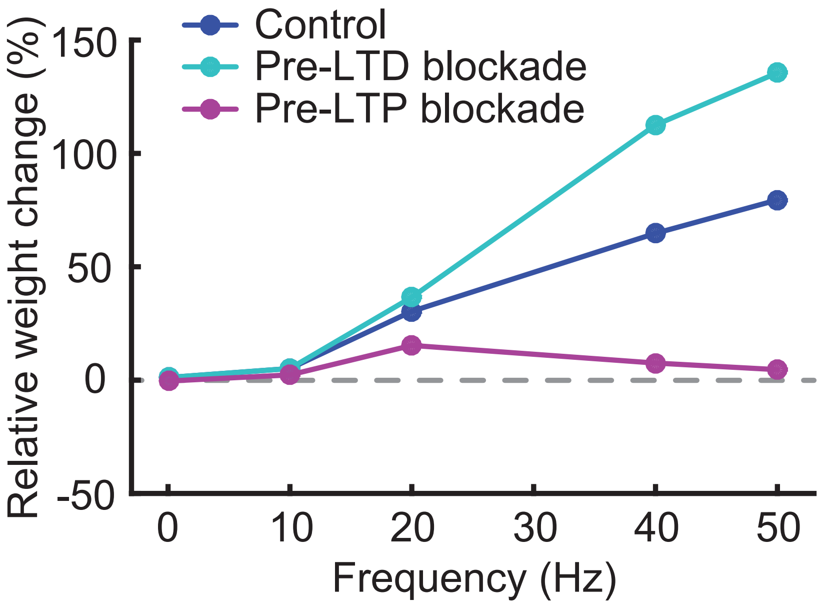

Figure S2. Blockade of presynaptic plasticity. Related to Figure 2. Model results are plotted as relative weight changes at a proximal location for different intra-burst frequencies. Pre- and postsynaptic bursts were shifted by +10 ms. The control condition (blue) equals model data of Figure 2B (right panel) in the main text, here compared to blockade of either pre-LTD (cyan) or pre-LTP (purple).

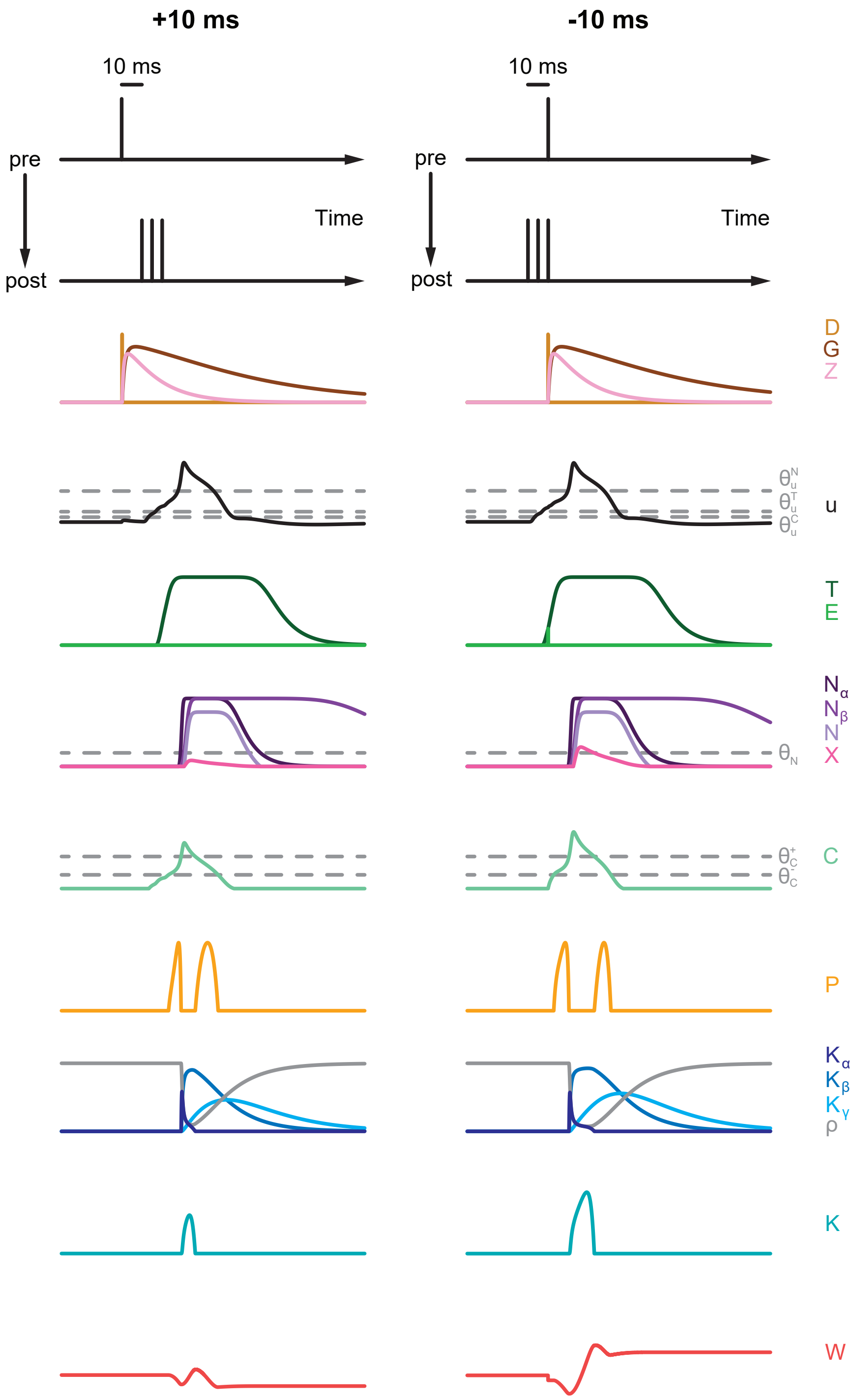

Figure S3. Model traces at distal locations in the burst-induced dendritic spike protocol. Related to Figure 4. Traces are drawn as according to Figure 1C in the main text. Note the delay between the somatic current injection (post trace, three spikes) and the peak of the dendritic spike at the synapse location, seen in the trace u.

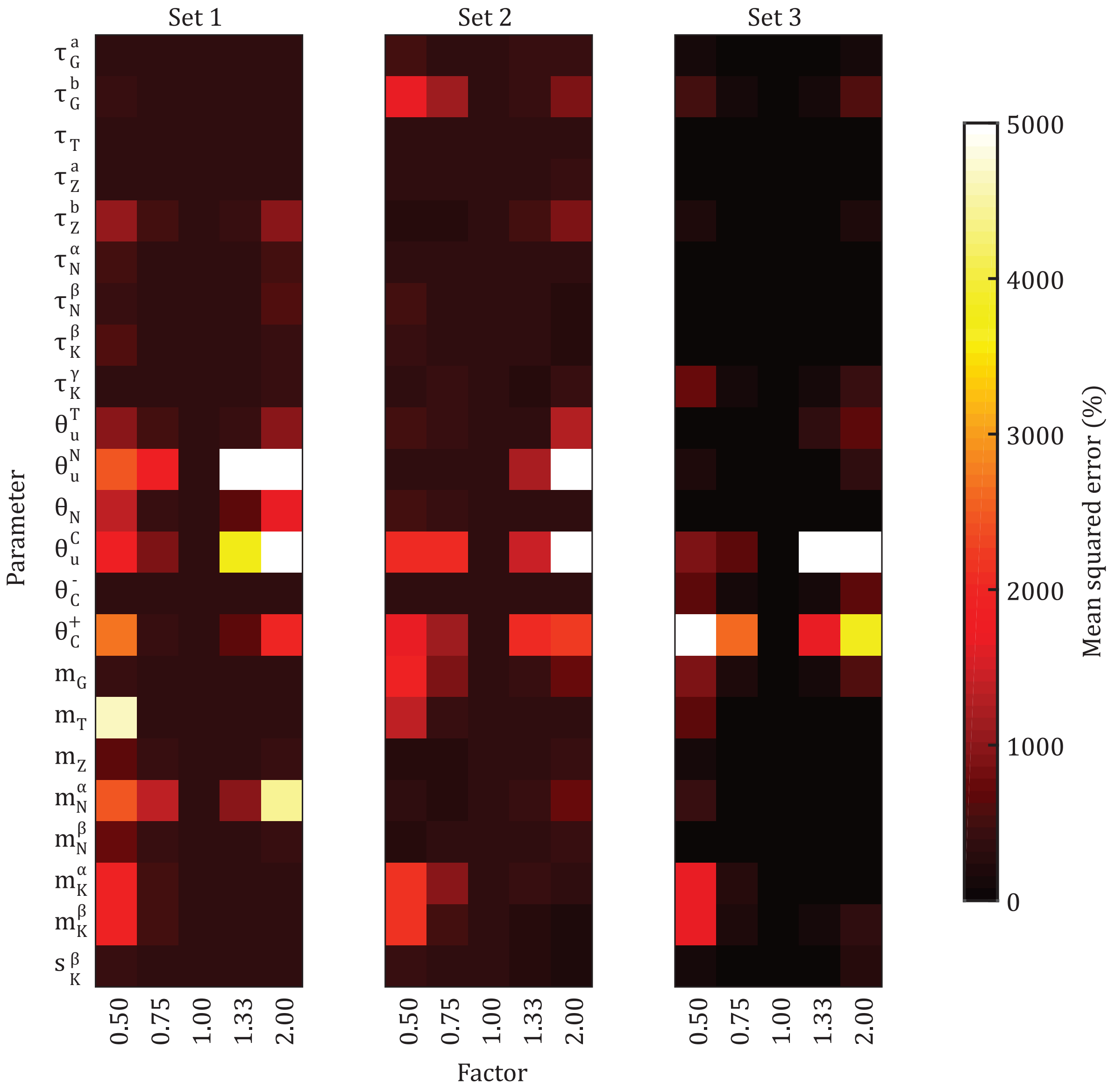

Figure S4. Sensitivity analysis. Related to STAR⋆Methods. Sensitivity matrices showing the mean squared errors (color-coded, percent weight change) compared to original experimental data following reductions or increases of core parameters (y-axis) by four different factors besides the original fit (x-axis). Sets indicate plasticity amplitudes (see Table S1), tested against the respective experimental data (set 1: Sjöström et al., 2001; set 2: Nevian & Sakmann, 2006; set 3: Letzkus et al., 2006).

Parameter	Set 1	Set 2	Set 3	Description
A_{pre}^{LTD}	$3 \cdot 10^{-3}$	$2.8 \cdot 10^{-3}$	$1.5 \cdot 10^{-3}$	Amplitude of pre-LTD
A_{pre}^{LTP}	$33 \cdot 10^{-4}$	$13 \cdot 10^{-4}$	$2.5 \cdot 10^{-4}$	Amplitude of pre-LTP
A_{post}^{LTD}	$3.6 \cdot 10^{-4}$	$3.6 \cdot 10^{-4}$	$7.5 \cdot 10^{-4}$	Amplitude of post-LTD
A_{post}^{LTP}	$20 \cdot 10^{-2}$	$57 \cdot 10^{-2}$	$7.8 \cdot 10^{-2}$	Amplitude of post-LTP
$\tau_G^a(ms)$		2		Rise time constant of G
$\tau_G^b(ms)$		50		Decay time constant of G
$\tau_T(ms)$		10		Low-pass filter time constant to calculate $\overline{T}$
$\tau_Z^a(ms)$		1		Rise time constant of Z
$\tau_Z^b(ms)$		15		Decay time constant of Z
$\tau_N^\alpha(ms)$		7.5		Low-pass filter time constant to calculate $\overline{N}_\alpha$
$\tau_N^\beta(ms)$		30		Low-pass filter time constant to calculate $\overline{N}_\beta$
$\tau_K^\beta(ms)$		15		Low-pass filter time constant to calculate $\overline{K}_\beta$
$\tau_K^\gamma(ms)$		20		Low-pass filter time constant to calculate K_γ
θ_u^T		-60		Threshold applied to u to calculate $\overline{T}$
θ_u^N		-30		Threshold applied to u to calculate $\overline{N}_\alpha$
θ_N		0.2		Threshold applied to $N_\alpha \cdot N_\beta$ to calculate N
θ_u^C		-68		Threshold applied to u to calculate C
θ_C^-		15		Lower threshold applied to C
θ_C^+		35		Upper threshold applied to C
m_G		10		Slope of the saturation function leading to G
m_T		1.7		Slope of the saturation function leading to T
m_Z		6		Slope of the saturation function leading to Z
m_N^α		2		Slope of the saturation function leading to N_α
m_N^β		10		Slope of the saturation function leading to N_β
m_K^α		1.5		Slope of the saturation function leading to K_α
m_K^β		1.7		Slope of the saturation function leading to K_β
s_K^β		100		Scaling factor for K_β

Table S1. Related to STAR⋆Methods. Parameters for the plasticity rule.
